# The RNA binding protein Arid5a is an activator of TNF signaling in rheumatoid arthritis

**DOI:** 10.1172/jci.insight.196411

**Published:** 2026-01-23

**Authors:** Yang Li, Ipsita Dey, Shachi P. Vyas, Alzbeta Synackova, Decheng Li, Erik Lubberts, Dana P. Ascherman, Peter Draber, Sarah L. Gaffen

**Affiliations:** 1 Division of Rheumatology & Clinical Immunology, Department of Medicine, University of Pittsburgh, Pittsburgh, Pennsylvania, USA.; 2 BIOCEV, First Faculty of Medicine, Charles University, Vestec, Czech Republic.; 3 School of Medicine, Tsinghua Medicine, Tsinghua University, Beijing, China.; 4 Erasmus MC University Medical Center, Department of Rheumatology, Rotterdam, Netherlands.; 5 Institute of Molecular Genetics of the Czech Academy of Sciences, Prague, Czech Republic.; 6 Deptartment of Immunobiology, University of Lausanne, Epalinges, Switzerland.

**Keywords:** Autoimmunity, Immunology, Arthritis, Chemokines, Cytokines

## Abstract

Rheumatoid arthritis (RA) is characterized by joint inflammation and bone erosion. Understanding cytokine pathways, particularly those targeting TNF, is crucial for understanding pathology and advancing treatment development. Arid5a is a noncanonical RNA binding protein (RBP) that augments inflammation through stabilizing proinflammatory mRNAs and enhancing protein translation. We examined published datasets for *ARID5A* in human RA blood, T cells, and synovial tissues. A stromal cell line, epithelial cells, and primary synovial fibroblasts were used to assess the effect of TNF on Arid5a expression, localization, and function. To determine how TNF induces Arid5a, WT or *Traf2^–/–^* stromal cells were treated with NIK or IKK inhibitors. To evaluate the necessity of Arid5a in arthritis progression, *Arid5a^–/–^* mice were subjected to collagen-induced arthritis. *ARID5A* was elevated in patients with RA and reduced by anti-TNF therapy. TNF upregulated Arid5a through the NF-κB1/TRAF2 pathway, causing cytoplasmic relocalization. Arid5a stabilized proinflammatory transcripts and enhanced expression of chemokines that drive RA. *Arid5a^–/–^* mice were resistant to collagen-induced arthritis correlating with reduced Th17 cells in synovial tissue. Thus, Arid5a serves as a newly recognized signaling intermediate downstream of TNF that is elevated in human RA and drives pathology in murine CIA, potentially positioning this RBP as a possible therapeutic target.

## Abstract 2

The cytokine TNF is a major driver of rheumatoid arthritis. We have identified a new mediator of TNF signaling through a noncanonical RNA binding protein, Arid5a. ARID5A mRNA is elevated in human RA tissues, and Arid5a-/- mice are resistant to collagen induced arthritis.

## Graphical abstract

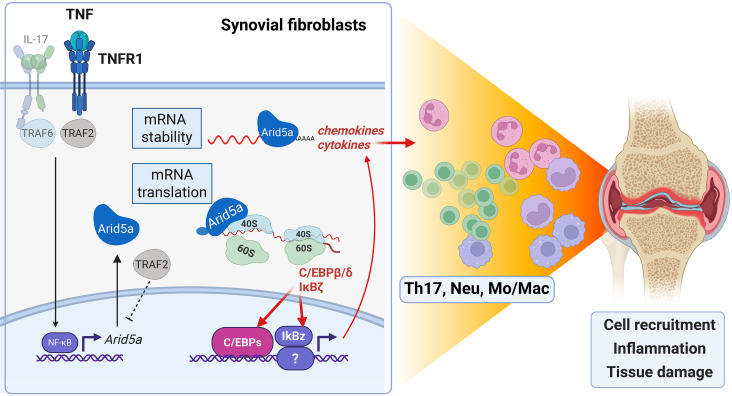

## Introduction

Rheumatoid arthritis (RA) is one of the most common autoimmune diseases of humans. RA primarily affects small joints in the hands and feet and has systemic effects as well, with multiple coexisting conditions and extraarticular manifestations. RA is 3 times more prevalent among women than men (1). Multiple susceptibility genes and epigenetic modifications are linked to arthritis pathogenesis, and behavioral risk factors such as smoking, obesity, and poor dental hygiene are contributors (2).

Although the causative etiology of RA remains uncertain, the advent of anticytokine biologic therapies revolutionized disease management. TNF inhibitors were the first and remain the most widely used class of anticytokine drugs for RA. Nonetheless, anticytokine drugs carry limitations such as variable or waning efficacy, infectious disease risk, substantial costs, and accessibility challenges. Consequently, there is an unmet need for alternative approaches that might reduce some of these barriers. Advances in understanding cytokine-mediated signaling pathways has demonstrated that pharmacological targeting of cytokine signaling is a fruitful strategy for rational intervention. For example, Janus kinase (JAK) inhibitors are widely used for autoimmune conditions, with distinct advantages over antibody-based biologics (3). Still, our understanding of the detailed signaling pathways that drive cytokine pathogenesis remain incomplete, and defining new cytokine signaling pathways could ultimately provide a basis for successful therapies.

Produced mainly by synovial macrophages, TNF activates strong proinflammatory signals in many cell types via its receptor TNFR1. TNF mediates signal transduction through a cascade centered around the multifunctional adaptor TRAF2. Studies of TNF signaling typically focus on activation of cell death and new gene induction via downstream transcription factors (TFs), such as NF-κB and AP-1 family members (4, 5). However, immune transcripts are often subject to posttranscriptional modes of regulation, particularly control of mRNA stability and translation (6). In that regard, TNF signals stabilize many transcripts operative in RA pathogenesis (7). For example, TNF induces miRs and the RNA binding protein (RBP) TTP (encoded by *ZFP36*), which determine the magnitude of inflammation by dictating the fate of TNF target genes (8).

AT-rich interaction domain protein 5a (Arid5a) is an RBP that controls RNA stability and translation of transcripts induced in response to several inflammatory stimuli relevant to RA, including TLR4 and IL-17 (9–11). In mice, Arid5a is required for pathogenesis of autoimmune models of multiple sclerosis (experimental autoimmune encephalomyelitis [EAE]), sepsis, and autoimmune glomerulonephritis (AGN) (12–14). Elevated levels of *ARID5A* are also linked to human crescentic GN (12). However, to date, there are no reported connections of Arid5a to TNF signaling events.

In this study, we demonstrate that Arid5a is elevated in the blood and synovial fluid of patients with RA and is reduced in patients treated with anti-TNF and anti–IL-6 biologics. TNF upregulates Arid5a expression through an IKK-mediated classical NF-κB pathway, while TRAF2 negatively regulates TNF-induced Arid5a expression. Arid5a induces expression of chemokines and cytokines operative in RA, prolonging transcript half-life and leading to impairment of immune cell transmigration. Arid5a-deficient mice are resistant to a collagen-induced model of inflammatory arthritis, despite normal generation of anti-collagen antibodies. Thus, we identify Arid5a as a new positively acting node in the TNF signaling pathway that promotes inflammatory arthritis.

## Results

### ARID5A expression correlates with RA severity.

**Figure 1:**
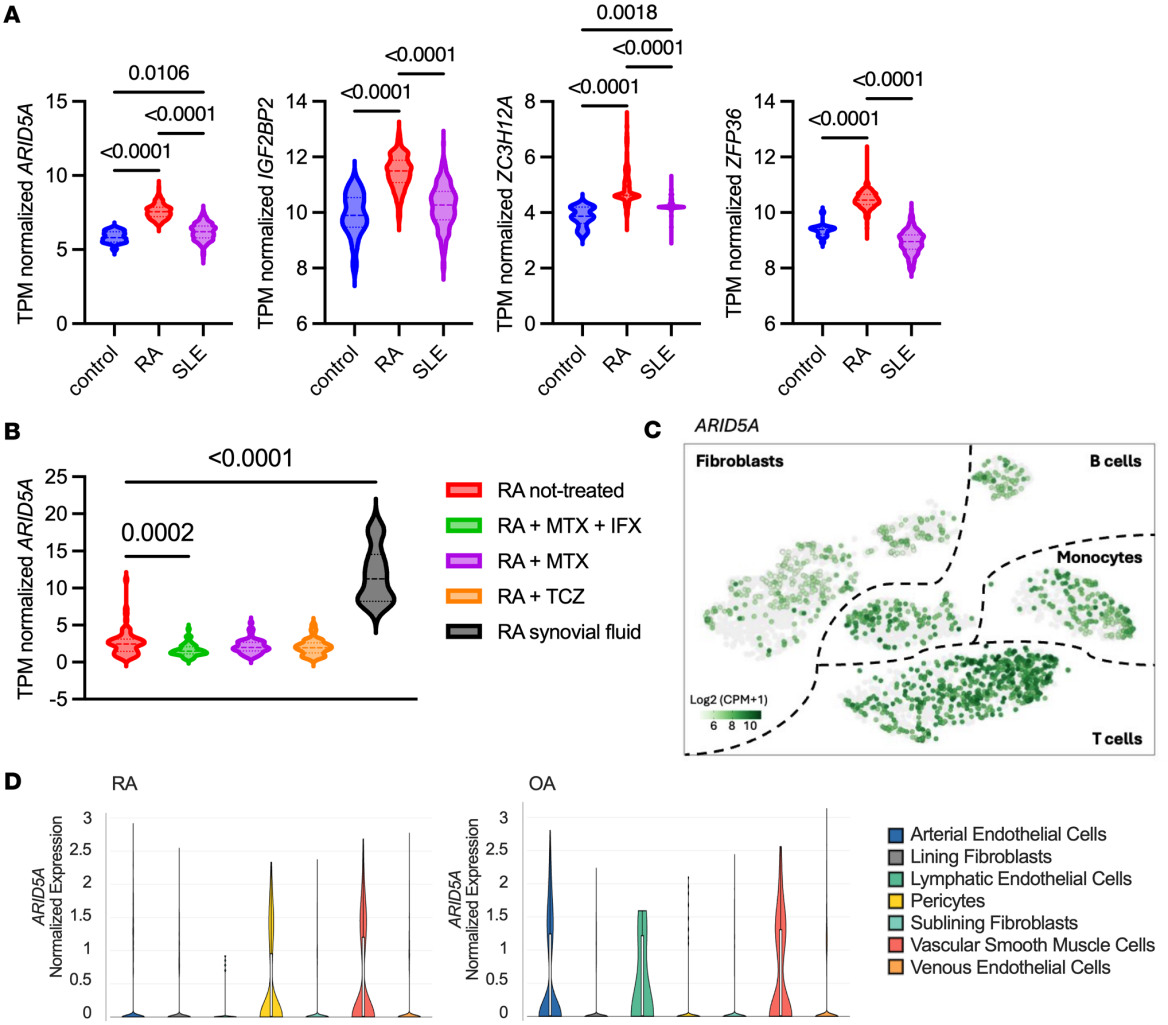
Expression of RNA binding proteins including ARID5A are elevated in RA blood and synovial tissue. Public datasets were examined for RBPs implicated in cytokine signaling. (**A**) Expression in RNA-Seq of whole blood; RA group is pooled data from RA-DMARD-IR and RA-TNF-IR cohorts. Analyzed by 1-way ANOVA with Tukey’s test (control *n* = 20; RA *n* = 493; SLE *n* = 292). (**B**) RNA-Seq of T cell data pooled from CD4^+^ and CD8^+^ groups treated with anti-TNF biologic (infliximab [IFX]) and methotrexate (MTX), MTX alone, or an anti–IL-6R biologic (tocilizumab [TCZ]). Levels in synovial fluid are shown for comparison. Analyzed by 1-way ANOVA with post-hoc Dunnett’s test (untreated *n* = 63; RA + MTX + IFX *n* = 63; RA + MTX *n* = 59; RA + TCZ *n* = 66; synovial fluid *n* = 11), comparing each group to untreated RA. TPM, transcripts per million reads. (**C**) *ARID5A* levels in RA versus OA in synovial tissue cell clusters. (**D**) *ARID5A* levels in RA versus OA in nonhematopoietic cells.

To better understand posttranscriptional pathways in RA, we interrogated public databases for expression of RBPs known to participate in TNF or related cytokine signaling cascades (15–18). *ARID5A* mRNA was elevated in whole blood samples from patients with RA compared with healthy controls or from patients with systemic lupus erythematosus (SLE) (Figure 1A). Expression of other RBPs was also increased, including *IGF2BP2* (IMP2), *ZC3H12A* (Regnase-1, MCPIP1), and *ZFP36* (Tristetraprolin [TTP]) (Figure 1A). Interestingly, expression of *ARID5A* in T cells was reduced in patients on combination therapy with methotrexate (MTX) and a TNF biologic inhibitor (infliximab [IFX]), MTX alone, or anti–IL-6R (tocilizumab [TCZ]) treatment (Figure 1B). *ARID5A* levels were even higher in synovial fluid samples from patients with RA than in T cells (Figure 1B). By contrast, *ZFP36* did not change with anti-TNF therapy, *IGF2BP2* was not expressed in T cells as previously reported (19), and *ZC3H12A* levels did not change regardless of treatment (Supplemental Figure 1A; supplemental material available online with this article; https://doi.org/10.1172/jci.insight.196411DS1). These data therefore point to a potential link between TNF signaling and Arid5a in the context of RA.

We examined *ARID5A* expression in a synovial tissue dataset that integrates single-cell transcriptomics and mass cytometry (20). In comparison with osteoarthritis (OA) control patients, *ARID5A* was elevated in RA synovial samples across cell types, including T cells, monocytes, and synovial fibroblasts (Figure 1C). *ZC3H12A* and *ZFP36* exhibited a similar pattern to *ARID5A*, while *IGF2BP2* was predominantly seen in fibroblasts (Supplemental Figure 1B). In a dataset that focused on nonhematopoietic cells in synovial tissue (21), *ARID5A* was more prominent in arterial endothelial cells and lymphatic endothelial cells in OA, whereas in RA, *ARID5A* was highly expressed in pericytes and vascular smooth muscle cells (Figure 1D). Both disease types expressed *ARID5A* in vascular smooth muscle cells. Collectively, these data indicate that *ARID5A* correlates with RA severity, with expression in both hematopoietic and nonhematopoietic compartments.

### TNF upregulates Arid5a.

**Figure 2:**
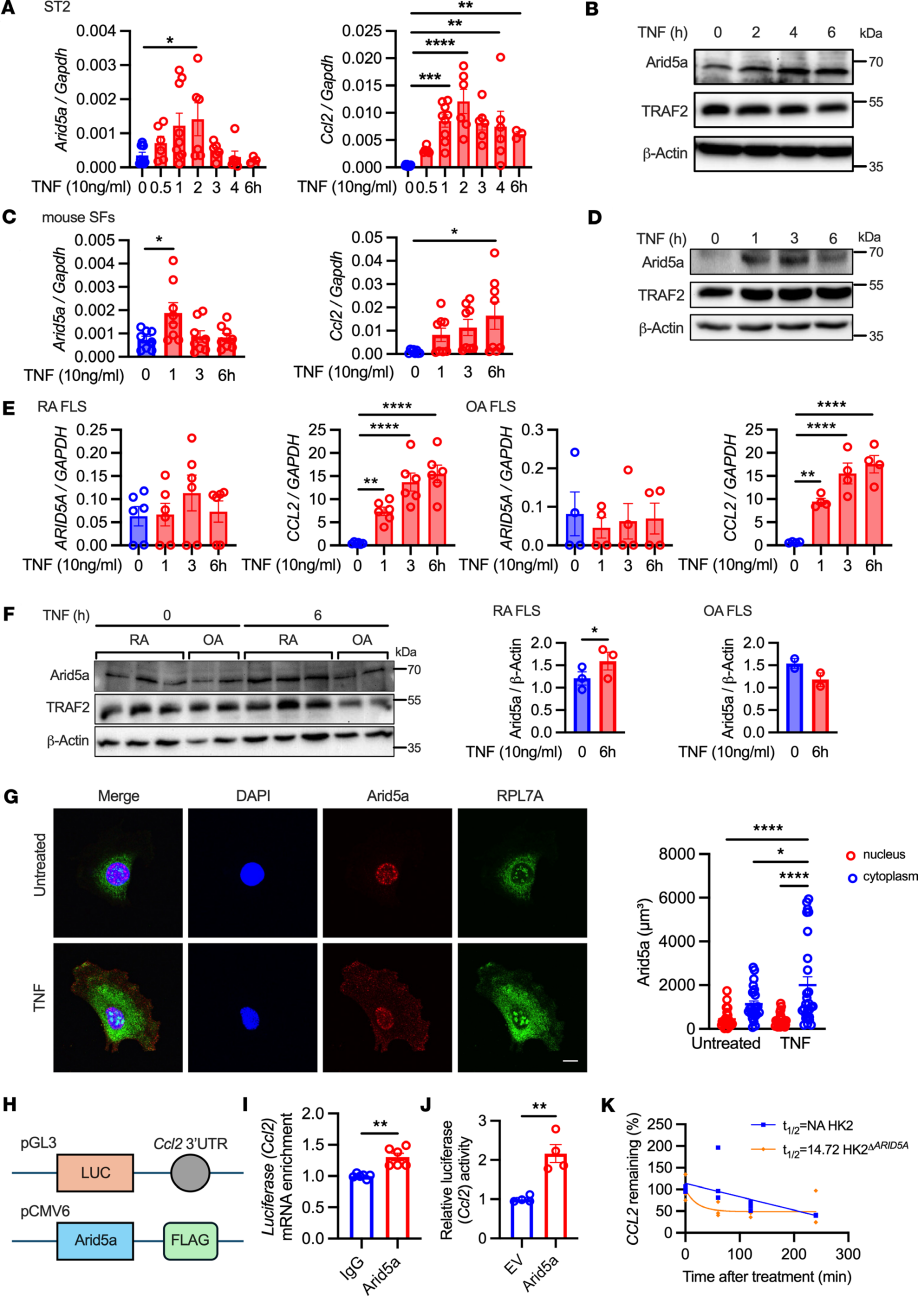
TNF induces Arid5a expression and nuclear export. (**A**) ST2 cells were treated with TNF and assessed by qPCR (*n* = 3–10). Analyzed by 1-way ANOVA with Dunnett’s test, comparing each time point to 0 hours. Data pooled from 3 experiments. (**B**) ST2 lysates were subjected to immunoblotting, representative of 3 experiments. (**C**) Mouse primary synovial fibroblasts were treated with TNF and mRNA assessed by qPCR (*n* = 8). Analyzed by 1-way ANOVA with Dunnett’s test, comparing to 0 hours. Data pooled from 4 mice. (**D**) Mouse primary synovial fibroblasts lysates was subjected to immunoblotting, representative of 4 experiments. (**E**) RA and OA FLS were treated with TNF and mRNA assessed by qPCR (*n* = 3 RA, *n* = 2 OA, with biological duplicates shown). Analyzed by 1-way ANOVA with Dunnett’s test, comparing to 0 hours. (**F**) RA and OA FLS were subjected to immunoblotting. Densitometry analysis. (**G**) Arid5a localization in ST2 cells treated with TNF for 3 hours. Scale bar:10 μm. Arid5a in nucleus vs. cytoplasm quantified by Arid5a volume per compartment from an individual cell (*n* = 25–27). Analyzed by 1-way ANOVA with Tukey’s test. (**H** and **I**) HEK293T cells were transfected with Arid5a-FLAG and pGL3-Luc fused to mouse *Ccl2*-3′ UTR. RIP was performed with anti-FLAG Abs or IgG and *Luc* assessed by qPCR (*n* = 6). Data pooled from 2 experiments. Analyzed by Student’s *t* test. (**J**) HK-2 cells were transfected with a *Luc*-*Ccl2* 3′UTR reporter and Arid5a-FLAG or empty vector (EV). Firefly luciferase activity was determined (*n* = 4). Data representative of 2 experiments. Analyzed by Student’s *t* test. (**K**) Half-life of *CCL2* in HK2 or HK2^ΔARID5A^ cells by 4sU labeling (*n* = 3). Analyzed by 1 phase decay best fit. NA, not applicable (half-life calculation is out of range). Data representative of 2 experiments. *P < 0.05, **P < 0.01, ***P < 0.001, ****P < 0.0001.

Since the proinflammatory receptor TNFR1 is ubiquitously expressed, TNF has the potential to act upon multiple cell types within the synovial compartment. Given the prominent expression of *ARID5A* in synovial cell types observed in the databases above and the coordinating role of synovial and stromal fibroblasts in potentiating arthritis (22), we next examined TNF signaling in various fibroblast/stromal cell types. In a murine stromal fibroblast cell line (ST2), *Arid5a* mRNA and protein were upregulated in response to TNF (Figure 2, A and B, and Supplemental Figure 2B). While *Arid5a* mRNA was induced transiently, elevated protein levels of Arid5a persisted for up to 6 hours. As expected, genes encoding inflammatory cytokines and chemokines were induced by TNF in this setting (*Il6*, *Ccl2*) as was *Zc3h12a* (Figure 2A and Supplemental Figure 2A). In mouse primary synovial fibroblasts (SFs), *Arid5a* mRNA and protein were similarly upregulated in response to TNF (Figure 2, C and D, and Supplemental Figure 2B). Chemokines (*Ccl2*, *Cxcl1*) and *Zc3h12a* were also induced by TNF (Figure 2C and Supplemental Figure 2C). In fibroblast-like synovial cells (FLS) obtained from patients with RA, TNF consistently upregulated Arid5a protein expression, though *ARID5A* mRNA only trended upward in response to TNF (Figure 2, E and F, and Supplemental Figure 2D). In contrast, FLS cells from patients with OA did not show Arid5a induction at the mRNA or protein level, though chemokines were induced, indicating that TNF signaling was intact in these cells. In ST2 cells, TNF stimulation caused Arid5a accumulation in the cytoplasm (Figure 2G), which is in agreement with prior findings that LPS and IL-17 promote nuclear export of Arid5a (12, 23).

Arid5a and other RBPs typically control stability of target mRNAs by binding to stem-loop structures in 3′ UTRs (6, 14). *Ccl2* was upregulated by TNF with similar kinetics as Arid5a, prompting us to ask whether Arid5a controls with the *Ccl2* 3′ UTR. To test this, FLAG-Arid5a was ectopically expressed in HEK293 cells with a *Ccl2*–3′ UTR sequence inserted downstream of luciferase (Figure 2H). Lysates were subjected to RNA immunoprecipitation (RIP) with anti-FLAG Abs or isotype controls. Indeed, the *Luc–Ccl2–3′ UTR* sequence was enriched after anti-FLAG (Arid5a) pulldown compared with IgG controls, consistent with direct binding of Arid5a to this 3′ UTR sequence (Figure 2I). Moreover, coexpression of Arid5a with *Luc–Ccl2–3′ UTR* enhanced expression of the luciferase reporter compared with an empty vector (Figure 2J). Note that a vector lacking the *Ccl2* 3′ UTR sequence was not transactivated in the presence of Arid5a, confirming that Arid5a exerts specific activities on the 3′ UTR rather than activating cryptic elements within the reporter (Supplemental Figure 2E). We next evaluated whether Arid5a mediates stabilization of human *CCL2* mRNA, taking advantage of a human epithelial cell line engineered to lack Arid5a (12). Based on a 4-thiouridine (4sU) RNA decay assay (24), *CCL2* half-life was reduced (Figure 2K), though another chemokine *CXCL1* was not affected (Supplemental Figure 2F). Collectively, these results indicate that Arid5a enhances the stability of *Ccl2* through binding the 3′ UTR, which is consistent with other unstable transcripts controlled by Arid5a such as *Il6* (11, 25, 26). Thus, Arid5a represents a potentially new posttranscriptional signaling intermediate in the TNF signaling cascade.

The rheumatoid synovium contains multiple cytokines that can promote cooperative or synergistic responses (7, 22, 27). TNF and IL-17 are well described to mediate such cooperative and/or overlapping signals, resulting in comparable, albeit not identical, downstream gene profiles (11, 12, 28). Additionally, there is crosstalk between transcriptional regulation mediated by TNF and posttranscriptional regulation mediated by IL-17, especially with respect to chemokine gene regulation (9, 29–32). However, TNF and IL-17 did not synergistically upregulate *Ccl2*, *Arid5a*, or *Zc3h12a*, although they exhibited a strong synergistic effect on *Il6* as previously described (33, 34) (Supplemental Figure 3A). Therefore, even though Arid5a is induced by both TNF and IL-17, this RBP is not regulated synergistically by these cytokines, nor are all its target genes controlled in a cooperative manner.

### TRAF2 negatively regulates Arid5a through the NF-κB pathway.

**Figure 3:**
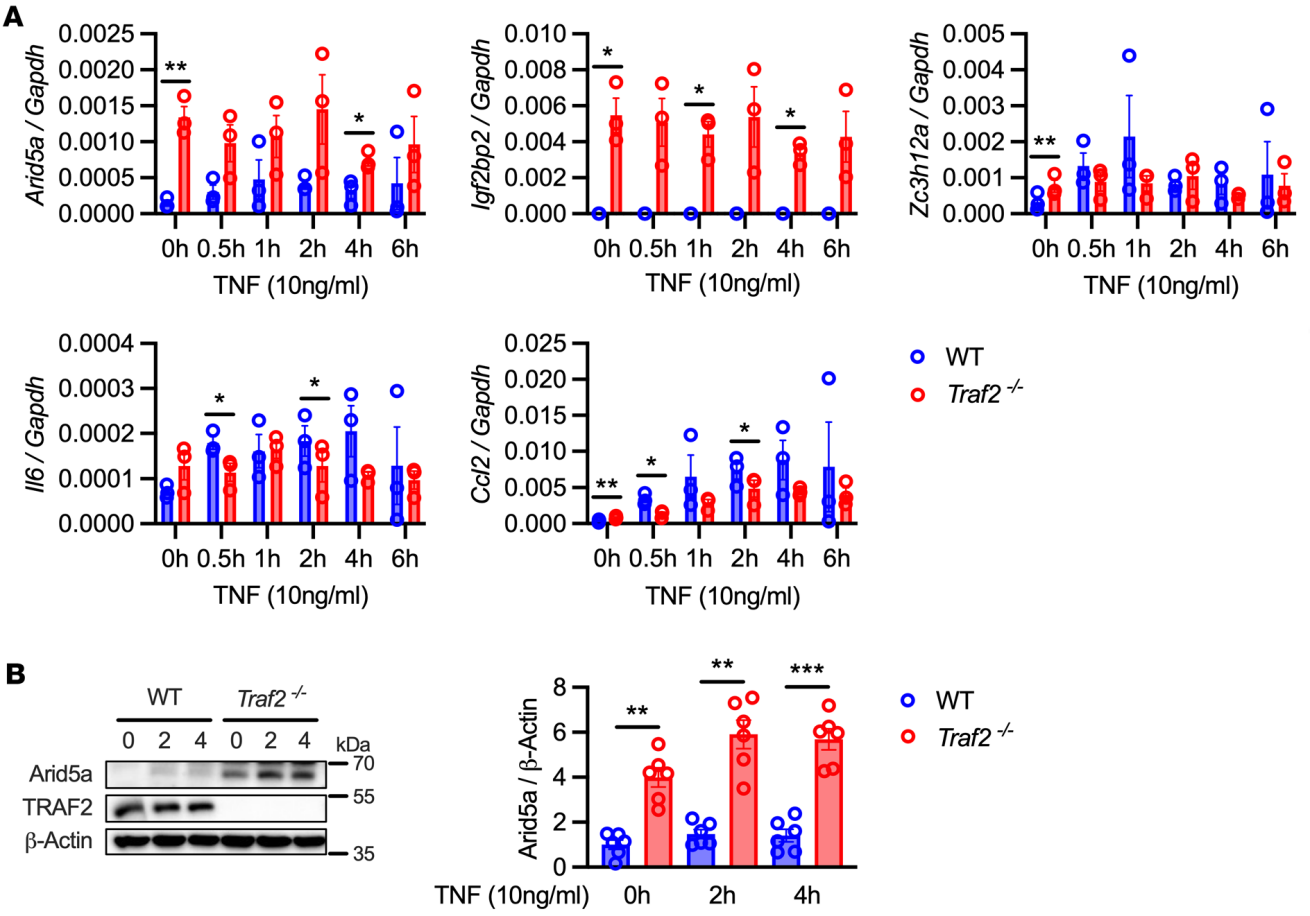
TRAF2 negatively regulates Arid5a. (**A**) WT or *Traf2^–/–^* ST2 cells were treated with TNF and indicated mRNAs assessed by qPCR (*n* = 3). Analyzed by Student’s *t* test, comparing WT or *Traf2^–/–^* ST2 cells for each time point. Data representative of 3 experiments. (**B**) WT or *Traf2^–/–^* ST2 cells were treated with TNF and lysates immunoblotted for Arid5a, TRAF2, or β-actin. Densitometry quantification at right, analyzed by Student’s t test, comparing WT or *Traf2^–/–^* ST2 cells for each time point (*n* = 6). Each symbol represents 1 sample. *P < 0.05, **P < 0.01, ***P < 0.001.

TRAF2 drives activation of both canonical and noncanonical NF-κB pathways, functioning upstream of the IKK complex or NIK (5, 35). To elucidate the role of TRAF2 in TNF-induced Arid5a expression, *Traf2^–/–^* ST2 cells (36) were stimulated cells with TNF over a time course of 6 hours. Surprisingly, TRAF2 appeared to negatively regulate *Arid5a* as well as *Igf2bp2* (encoding IMP2) at both basal and TNF-induced states (Figure 3A). At the protein level, ST2 cells lacking TRAF2 exhibited marked upregulation of Arid5a and IMP2 (Figure 3B and Supplemental Figure 3B). However, this was not the case for all TNF-induced target genes, including for *Zc3h12a, Il6*, and *Ccl2* (Figure 3A). A similar pattern of inhibitory activity was observed in the context of IL-17 signaling, as ST2 cells lacking TRAF2 showed enhanced induction of *Arid5a* and *Igf2bp2* in response to IL-17 (Supplemental Figure 3C). As previously shown, TRAF2 was required for IL-17 induction of *Il6* and *Cxcl1* (11, 37) (Supplemental Figure 3C). TRAF2 deficiency caused increased Arid5a and IMP2 proteins but not other IL-17–associated TFs (C/EBPs, IκBζ) (Supplemental Figure 3D).

### TRAF2 inhibits Arid5a through IKK but not NIK signaling.

**Figure 4:**
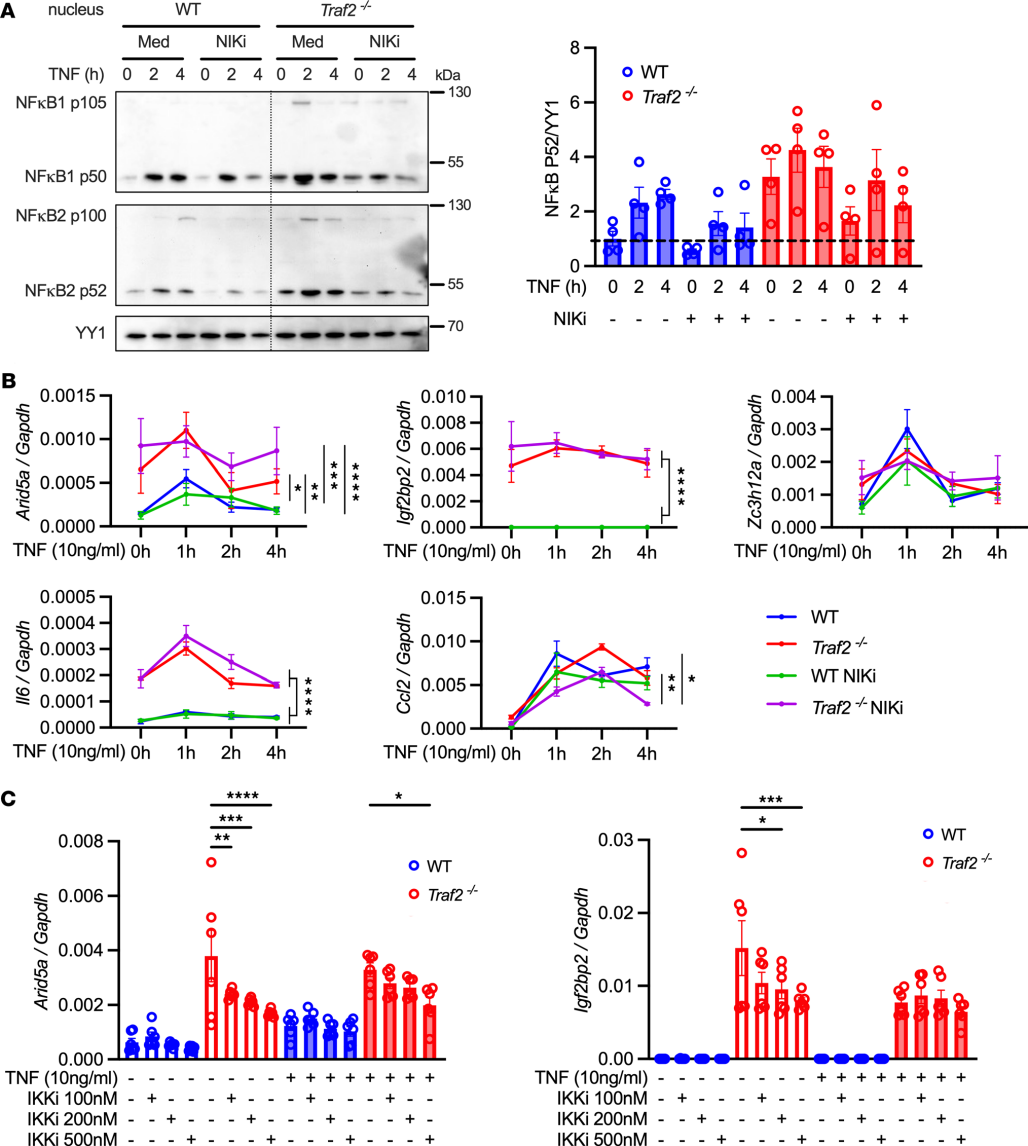
TRAF2 inhibits Arid5a through IKK-dependent NF-κB signaling. WT or *Traf2^–/–^* ST2 cells were pretreated with NIKi for 16 hours followed by TNF + NIKi for the indicated times. (**A**) Nuclear lysates were immunoblotted for NF-κB1, NF-κB2, or YY1, representative of 4 experiments. Densitometry is shown at right (*n* = 4). Each symbol represents 1 sample. (**B**) Indicated genes assessed by qPCR. Each symbol represents the mean ± SEM of 3 individual samples. (**C**) WT or *Traf2^–/–^* ST2 cells were pretreated with IKKi for 20 hours, treated with TNF + IKKi for 1 hour, and genes assessed by qPCR (*n* = 6). Analyzed by 1-way ANOVA with Šídák’s test comparing control to IKKi treatment. Data are representative of 3 experiments. *P < 0.05, **P < 0.01, ***P < 0.001, ****P < 0.0001.

TRAF2 negatively regulates noncanonical NF-κB by inhibiting NIK, resulting in enrichment of NF-κB and ERK pathways (38). The ENCODE ChIP-Seq dataset indicates that RELB and NF-κB2 recognition elements are present in the *ARID5A* proximal promoter (Supplemental Figure 4A) (39). To determine whether TRAF2 negatively regulates Arid5a through the noncanonical NF-κB pathway, WT or *Traf2^–/–^* ST2 cells were treated with a NIK inhibitor. As expected, NF-κB2 p52 levels were higher in the nucleus of *Traf2^–/–^* compared with WT cells, regardless of TNF stimulation. Conversely, NF-κB2 p52 levels were decreased with NIK inhibition (Figure 4A). However, *Arid5a* levels were not affected by NIK inhibitor treatment, nor were *Igf2bp2*, *Zc3h12a*, *Il6*, and *Ccl2* (Figure 4B). Moreover, the NIK inhibitor did not affect the elevated *Arid5a* seen in TRAF2-deficient cells, supporting the idea that the noncanonical NF-κB pathway does not contribute to *Arid5a* expression. There was also no effect of the NIK inhibitor on genes induced by IL-17, although a role for this kinase has been suggested in IL-17 signalling(40) (Supplemental Figure 4, B and C).

To determine whether IKKs are required for the inhibitory properties of TRAF2 with respect to Arid5a induction, we exploited the differential inhibitory properties of pharmacological IKK blockers. IKK inhibitor VII exhibits selective inhibition of IKK through an ATP-competitive mechanism (IKK2 IC_50_ = 40 nM, IKK complex IC_50_ = 70 nM, IKK1 IC_50_ = 200 nM). *Arid5a* exhibited a dose-dependent decrease in *Traf2^–/–^* ST2 cells upon IKK inhibitor treatment, irrespective of TNF (Figure 4C). *Igf2bp2*, *Zc3h12a*, *Il6*, and *Ccl2* exhibited similar trends (Figure 4C and Supplemental Figure 5). Collectively, these data suggest that TRAF2 negatively regulates Arid5a in an IKK-dependent manner and does not rely on a NIK-mediated NF-κB2 pathway.

### Arid5a^–/–^ mice are resistant to CIA.

**Figure 5:**
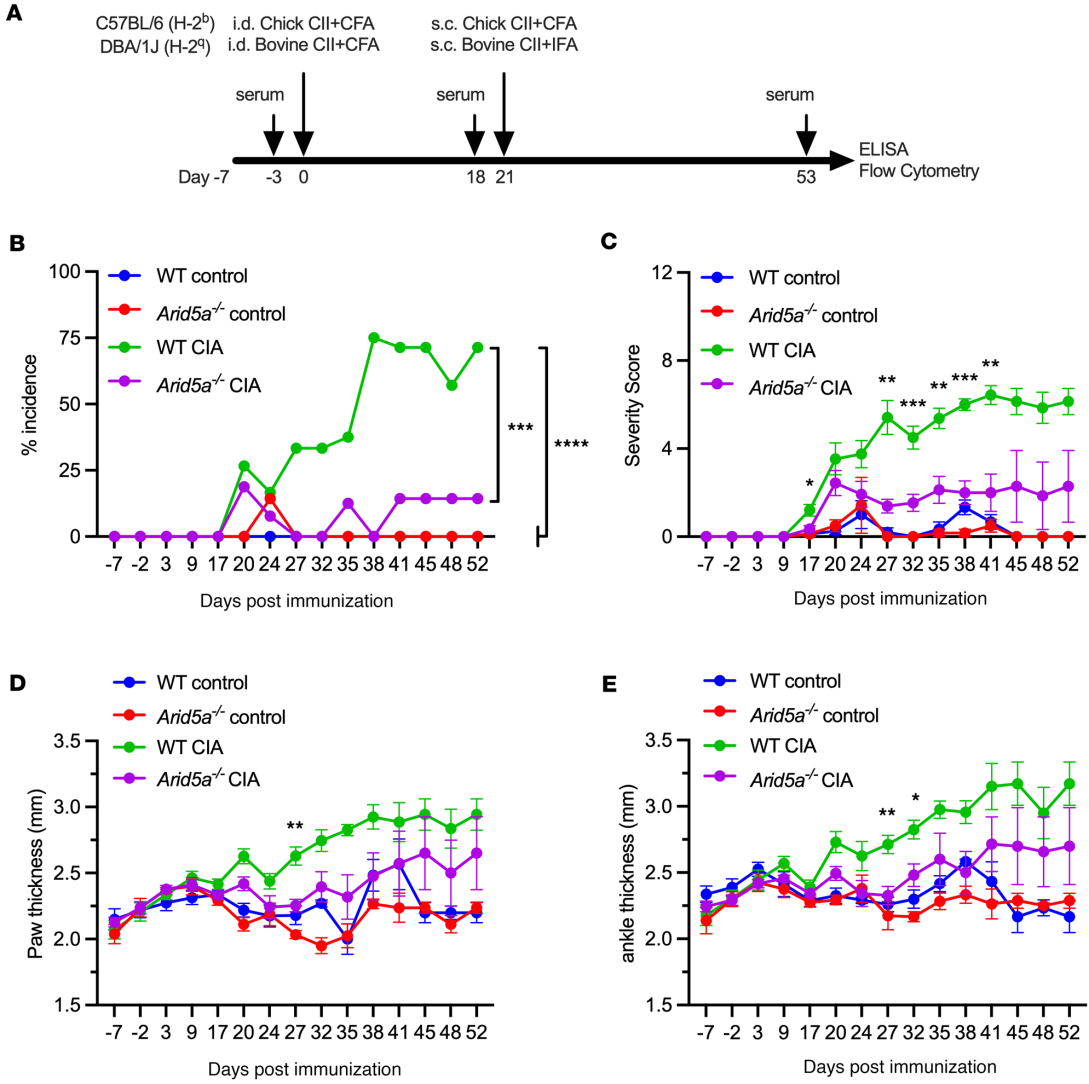
Arid5a is required for inflammatory pathogenesis in collagen-induced arthritis. (**A**) Timeline of CIA. (**B**) Incidence of CIA in C57BL/6 WT or *Arid5a^–/–^* mice, presented as percent of mice with symptoms (Supplemental Figure 6) (44). Analyzed by 1-way ANOVA with Tukey’s test for multiple comparisons. (**C**–**E**) Severity scores, paw and ankle thickness in WT and *Arid5a^–/–^* mice. Analyzed by 2-way ANOVA with Tukey’s test for multiple comparisons in each time point (WT control = 8, WT CIA = 15, *Arid5a^–/–^* control = 8, *Arid5a^–/–^* CIA = 18), pooled from 3 independent experiments. Asterisk denotes statistically significant difference between WT and *Arid5a^–/–^* mice. *P < 0.05, **P < 0.01, ***P < 0.001, ****P < 0.0001.

Given these connections of Arid5a to TNF and human RA, we hypothesized that Arid5a plays a causative role in autoimmune-induced inflammatory arthritis using *Arid5a^–/–^* mice (13) subjected to collagen-induced arthritis (CIA). Pathogenesis in this model is TNF dependent and is widely used to understand RA disease drivers (41, 42). WT or *Arid5a^–/–^* mice (C57BL/6, H-2^b^) were immunized with chicken collagen Type II (CII) in CFA on days 0 and 21 (43). Over the course of 60 days, joint inflammation and arthritis severity were scored in a single-blinded manner (Figure 5A and Supplemental Figure 6) (44). As a positive control, CIA was induced in the susceptible mouse strain DBA/1 (H-2^q^), which developed disease after the first CII immunization with an incidence approaching 100% (44–46) (Supplemental Figure 7, A and B). C57BL/6 WT mice developed arthritis symptoms more slowly, after the second CII immunization, with an incidence of ~75% (Figure 5B). The arthritis severity scores and incidence were lower in *Arid5a^–/–^* mice compared with WT controls, indicating marked delay of CIA as a result of *Arid5a* deficiency (Figure 5, B–E, and Supplemental Figure 7, C–E). These data confirm that Arid5a is not only expressed in RA but promotes inflammatory pathology of autoimmune arthritis.

**Figure 6:**
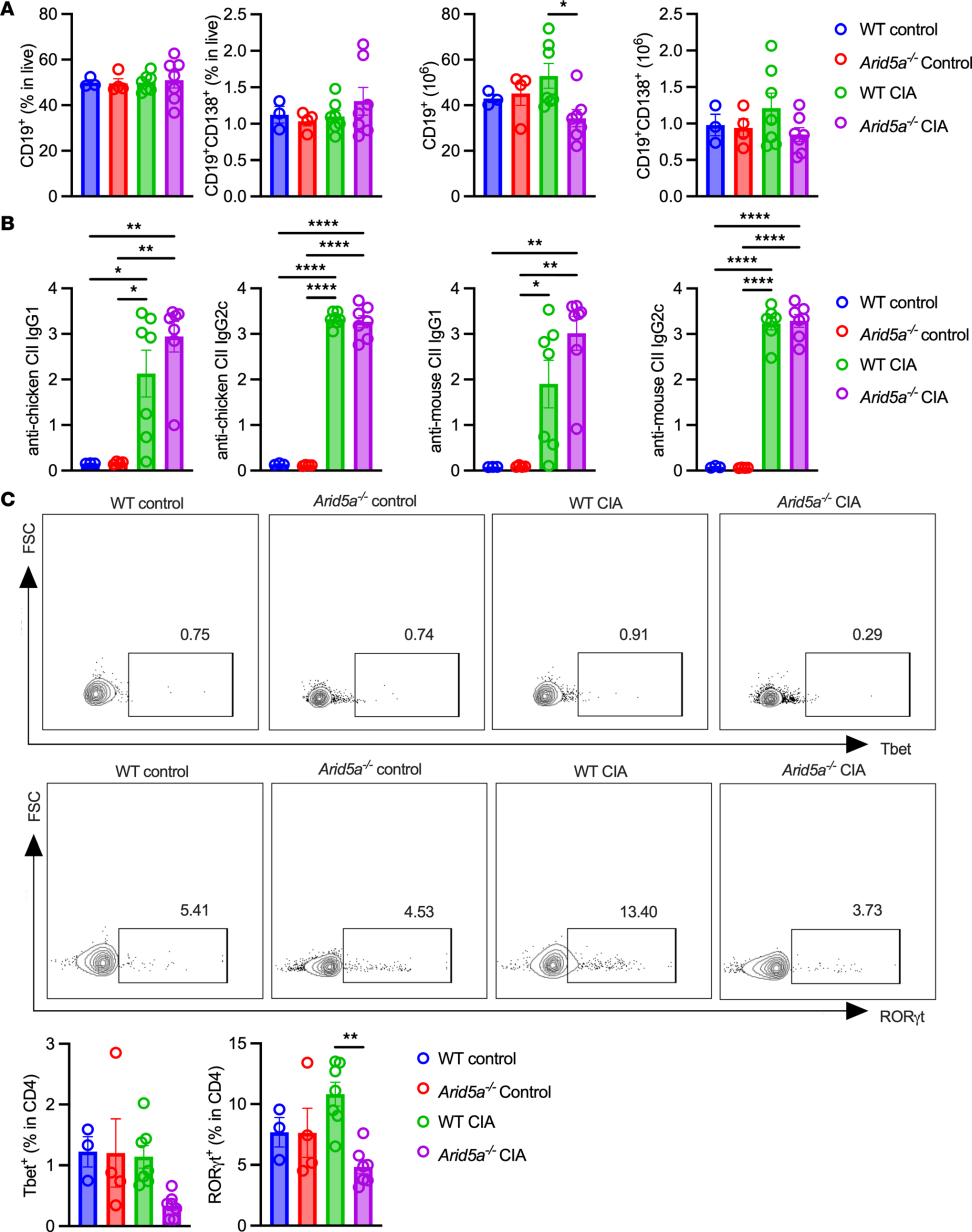
Arid5a is required to drive Th17 cells but not B cell/IgG responses in CIA. WT or *Arid5a^–/–^* mice were subjected to CIA and evaluated on day 53 (WT control = 3, WT CIA = 7, *Arid5a^–/–^* control = 4, *Arid5a^–/–^* CIA = 7). (**A**) Splenocytes were assessed for percentages and numbers of total and plasma B cells. (**B**) Serum levels of anti-chicken and anti-mouse collagen-II. (**C**) Representative plots of Tbet^+^ and RORγt^+^ cells in synovial tissue. Percentages of Tbet^+^ and RORγt^+^ in CD4^+^ cells in synovial tissue samples. Analyzed by 1-way ANOVA with Tukey’s test. Each symbol represents 1 mouse. *P < 0.05, **P < 0.01, ****P < 0.0001.

Arid5a is expressed ubiquitously, including in B cells, and generation of anti-CII antibodies is essential for arthritis in CIA (47). Overall changes in total B cell numbers, measured at day 53, were modest, with a mild impairment in *Arid5a^–/–^* mice and no statistically significant changes in plasma cell numbers (Figure 6A). There were also no differences between WT and *Arid5a^–/–^* mice in terms of antibody generation against the immunogen (chicken CII) and autoantibodies to mouse CII, measured prior to immunization (day –3) or at days 18 and 53 (Figure 6B and Supplemental Figure 8A). Th17 cells are key mediators in CIA, especially for joint destruction, and their numbers are largely independent of anti-CII antibody levels (47, 48). While inflammatory cell numbers were low at this chronic time point (day 53), infiltrating CD4^+^ T cells were present in the synovium (Figure 6C and Supplemental Figure 8B). RORγt^+^ cells were increased in WT CIA synovial tissue compared with *Arid5a^–/–^* mice (Figure 6C), consistent with a known role for Arid5a in Th17 cell generation (25), and Tbet^+^ cells trended downward in *Arid5a^–/–^* mice (Figure 6C).

To determine whether Arid5a regulates TNF-induced chemokine expression, ST2 cells were engineered to lack Arid5a by CRISPR/Cas9. *Arid5a*-deficient ST2 cells showed impaired CXCL1 production after TNF stimulation (Supplemental Figure 8C). Conditioned supernatants from TNF-treated ST2 cells with or without Arid5a were used in a trans-well assay to evaluate chemoattraction of CD45^+^ cells. As shown, total CD45^+^ cells, CD11b^+^ myeloid cells, CD4^+^ T cells, and neutrophils showed reduced migration in response to conditioned supernatants derived from TNF-treated ST2 cells lacking Arid5a (Supplemental Figure 8, D and E). Proinflammatory monocytes also demonstrate a decreasing trend in *Arid5a^–/–^* ST2 cells (Supplemental Figure 8E). Thus, while Arid5a is dispensable for the B cell response leading to autoantibody generation, both Th17 cell differentiation and production of immune-recruiting chemokines is impaired in mice lacking Arid5a.

## Discussion

RA affects an estimated 0.5%–1% of the global population and disproportionately occurs in women (49). Although anticytokine biologics have been transformative for this condition, they come with high medical costs, side effects, and incomplete and/or waning efficacy. Understanding the targets of cytokines that drive disease has potential to reveal new targets that might be exploited. Arid5a represents an attractive candidate in this sense for several reasons (50). Arid5a functions downstream of multiple inflammatory stimuli/cell types operative in RA, including TLR4 in macrophages, IL-17R in fibroblasts, and the TCR in CD4^+^ T cells (10, 51). Thus, an Arid5a-targeting approach would serve to suppress a variety of immune activities. Although this could also result in increased undesired side effects, we showed that loss of Arid5a in mice has no effect on susceptibility to opportunistic fungal infections (*Candida albicans*) that may be associated with anti-Th17/IL-17 and/or TNF blockade (13, 52, 53).

Our analysis of clinical datasets revealed that several RBPs linked to IL-17 or TNF signaling are elevated in RA, including mRNAs encoding Arid5a, TTP, Regnase-1, and IMP2. Of the RBPs examined, *ARID5A* was the only one whose expression correlated with TNF inhibition, although the database in question only showed expression data for T cells. It was reported that TCZ (targeting IL-6R) reduced Arid5a expression in RA (54). Arid5a directs lineage differentiation to disease-associated Th1 and Th17 cell types through stabilization of *Tbx1*, *Il6*, *Stat3*, and *Ox40* (11, 14, 25, 54–56). Arid5a also acts in macrophages and fibroblasts to promote cytokine/chemokine expression in response to TLR4, IL-17, and other stimuli (10). Here, we additionally noted that human pericytes, vascular mural cells that line capillaries, appear to be expressors of *ARID5A* in RA cohorts. Pericytes express TNFR1 as well as chemokines linked to RA inflammation (57, 58); it is plausible that Arid5a in pericytes may drive recruitment of inflammatory cells with destructive capacity to the synovium.

Arid5a is required for pathology in EAE (13, 14), and it has been assumed that Arid5a acts in T cells to mediate disease. However, importantly, this has not been experimentally established. In a murine model of autoimmune glomerulonephritis, Arid5a activity was required only in the nonhematopoietic compartment (mainly renal epithelial cells), likely due to the strong IL-17–driven component of this disease model (12, 59). Interestingly, here we saw a regulatory role for Arid5a in prompting RORγt expression in synovial CD4^+^ T cells, though whether Arid5a acts in a T cell–intrinsic manner is unknown. Thus, in RA, it is probable that Arid5a operates in a variety of cell types, and elucidating those that are most important in any given setting will be needed to understand the biology of this condition.

Studies of cytokine signaling often center around activation of TFs such as NF-κB or STATs, which in turn trigger transcription of inflammatory mRNAs. Comparatively few studies focus on posttranscriptional mechanisms that dictate the fate of such mRNAs or the proteins they encode. Arid5a was originally described as part of a family of DNA-binding TFs, with the capacity to stimulate chondrocyte differentiation (60–62). Arid5a also has mRNA binding capacity and enhances transcript half-life through binding AU-rich 3′ UTR elements (10, 11, 14, 56, 63). Therefore, Arid5a binds to chemokine and cytokine transcripts implicated in RA, including *Il6*, *Cxcl1*, *Ccl2*, and *Ccl20* (12). Adding to its complexity, Arid5a also promotes RNA translation through associations with the 40S ribosome and components of the translation initiation complex (12). In this way, Arid5a enhances protein levels of TFs such as C/EBPβ/δ and IκBζ that further increase the magnitude of inflammation (11, 12). Hence, Arid5a is a feed-forward signaling activator that promotes crosstalk between new gene transcription and posttranscriptional gene control.

There are intricate connections in cytokine signaling circuitry concerning Arid5a. In its capacity as an RBP, Arid5a counteracts an inhibitory endoribonuclease Regnase-1 (also known as MCPIP1, encoded by *Zc3h12a*). Arid5a and Regnase-1 compete for occupancy at a stem-loop structure in the *Il6* 3′ UTR, functionally offsetting each other’s actions (11, 14). Arid5a binds to *Zc3h12a* and thereby mediates Regnase-1 regulation (12). Despite suppressive functions that constrain cytokine-induced inflammation, Regnase-1 is often elevated in autoimmunity, as observed in psoriatic skin and demonstrated here in blood from patients with RA (64–66). In an analogous manner, TTP is a feedback inhibitor of TNF signaling (67), yet its mRNA (*ZFP36*) is elevated in RA. However, we did not see alterations in *ZFP36* expression upon anti-TNF therapy. Another RBP operative in TNF and IL-17 posttranscriptional signaling is *IGF2BP2* (encoding IMP2), a reader of N6-methyladenosine (m^6^A) (51, 68). IMP2 promotes expression of inflammatory genes in the IL-17 and TNF pathways including CEPBβ/δ (32, 51, 68), though IMP2 has also been reported to suppress inflammation in RA by regulating stability of the antioxidant gene *GSTM5* (69). Thus, control of gene expression in response to cytokines in RA is directed through highly complex interactions of various RBPs.

With respect to TNF receptor activation, TRAF2 enables assembly of a multiprotein signalosome complex including E3 ubiquitin ligases (cIAP1, cIAP2, LUBAC), which form nondegradative polyubiquitin linkages that in turn recruit IKK and TAK1-TAB complexes. TRAF2 does not have intrinsic E3 activity, so its effects are likely mediated by other Ub ligases — for example, cIAPs. These processes lead to activation of classical NF-κB and MAPK cascades. TNF also induces cell death via a RIPK1-dependent pathway. In addition, TRAF2 forms a cytoplasmic TRAF3-TRAF2-cIAP complex that constitutively degrades NIK and restricts noncanonical NF-κB signal transduction (4, 5). Hence, TRAF2 has dual roles, both promoting induction of inflammatory cytokines and negatively regulating Arid5a and other RBPs. Orchestrating these transcriptional activation events is essential for the outcome of the numerous biological activities induced by TNF, and we now show that Arid5a is a player in this regard.

Our findings in CIA demonstrate a role for Arid5a as a driver of arthritis. Despite expression in B cells, Arid5a did not affect generation of anti-type II collagen autoantibodies. The lack of correlation between IgG levels and CIA severity may be attributed to several factors, including the dominance of cell-mediated immunity, IgG subclass heterogeneity, glycosylation, immune complex dynamics, regulatory mechanisms, temporal discrepancies, antigen specificity, model-specific factors, and methodological considerations (70–73). We link Arid5a to Th17 cell production in CIA, consistent with observations that Arid5a drives efficient Th17 differentiation (25), and a critical role for IL-17–producing CD4^+^ T cells has been demonstrated in development of CIA (74). Therefore, controlling Th17 differentiation and activity of Th17 cells is likely an important component in the CIA-refractory phenotype seen in Arid5a-deficient mice.

Inflammatory cytokines such as TNF, IL-6, and IL-17 — all of which involve Arid5a signaling — provided impetus for the present study. While we postulate that the arthritogenic effects of Arid5a are due to its combined effects on immune cells, we cannot rule out a more direct role on bone cells. Bone turnover is regulated by the coordinated actions of cells that degrade bone (osteoclasts, which are hematopoietic and closely related to macrophages) and cells that coordinate bone repair and replacement (osteoblasts, which are mesenchymal in origin similar to fibroblasts) (75). While no studies have examined Arid5a in specific bone lineages, in its capacity as a TF, Arid5a was linked to chondrocyte differentiation through interactions with Sox9 (61). Nonetheless, there are not obvious bony defects in *Arid5a^–/–^* mice (e.g., there are no apparent issues with tooth eruption, which requires functional osteoclasts, nor do mice manifest growth retardation or other deficiencies).

In summary, these studies show that Arid5a is elevated in human RA, is induced by the arthritogenic cytokine TNF, and is causative for disease in a standard model of murine autoimmune arthritis. Exploiting RNA and RBPs pharmacologically is of increasing interest (76, 77). Indeed, chlorpromazine was reported to block Arid5a induction of *Il6* through interference with its capacity to bind RNA (50), though this phenomenon has not been examined in depth. Nonetheless, we still have not fully tapped the potential of cytokine signaling pathways, particularly with regard to posttranscriptional mechanisms of gene control.

## Methods

### Sex as a biological variable.

RA is approximately 3 times more prevalent in women. This study examined male and female animals, and similar findings are reported for both sexes. FLS were from both male and female with RA or OA, which showed similar findings.

### Study design.

The objective of this study was to determine the role and mechanistic basis of Arid5a in RA. We examined public databases of gene expression in patients with RA, comparing to OA and SLE as controls. We used cell culture studies to examine TNF signaling and a collagen-induced mouse arthritis model in *Arid5a*^–/–^ mice. Sample sizes were determined by power analyses from pilot studies or previously published data. No data were excluded. Mice were assigned randomly to experimental cohorts. Investigators were not blinded to group comparisons except for in vivo model CIA severity score assessments and ankle/paw monitoring, which was assessed using a single-blind method. Endpoints were selected based on experience or reports from prior studies.

### Mice.

*Arid5a^–/–^* mice were created as described (13) and are available at The Jackson Laboratory. C57BL/6 WT mice obtained from breeding served as littermate controls. DBA/1J mice were from The Jackson Laboratory. All mice were age and sex matched.

### CIA.

CIA model was performed as described (44–46). Briefly, for DBA/1J mice, 100 μg bovine type II collagen (Chondrex, #20012) in complete Freund’s adjuvant (CFA) (5 mg/mL; Chondrex, #7023) was injected i.d. in tail skin. On day 21, mice were immunized with 100 μg bovine type II collagen (Chondrex, #20012) in incomplete Freund’s adjuvant (Chondrex, #7002) by 2 s.c. injections at the left and right upper back. For C57BL/6, 100 μg chick type II collagen (Chondrex, #20012) in CFA (5 mg/mL; Chondrex, #7023) was injected i.d. in the tail. On day 21, mice were s.c. immunized with 100 μg chick type II collagen (Chondrex, #20012) in CFA at the upper back. Anti-chicken or anti-mouse IgG1 and IgG2c were measured by ELISA (SouthernBiotech, 5300-05B) on days –3, 18, and 53.

### Cell culture, cytokines, and inhibitors.

HK-2 cells (ATCC) were cultured in Dulbecco’s MEM/F12 (Gibco) with antibiotics and 10% FBS. HK-2*^ΔARID5A^* cells were described (12). ST2 cells were cultured in α-MEM (Sigma Aldrich) with L-glutamine, antibiotics and 10% FBS. *Arid5a*^–/–^ ST2-KO cells were prepared using CRISPR-Cas9. The web tool CHOPCHOP (78) was used to select target site (TTTTCATGCGTACCCCACCG), which was inserted into the pSpCas9(BB)-2A-GFP (PX458) plasmid (Addgene 48138). ST2 cells were transfected with Lipofectamine 2000 (Invitrogen) and GFP^+^ cells sorted on FACSAria Fusion (BD Biosciences). Single-cell clones were tested by immunoblotting and sequencing. *Traf2^–/–^* ST2-KO cells were described (36). HEK293T cells were cultured in α-MEM (Sigma Aldrich) with L-glutamine, antibiotics, and 10% FBS.

To obtain primary mouse SFs, synovial tissues were digested by 5 mg/mL of collagenase type IV (Sigma) for 60 minutes and filtered in a 70 μm cell strainer (79). Cells were cultured in Dulbecco’s MEM (Gibco) with 10% FBS and antibiotics. Experiments were performed on passage 3. RA or OA FLS were obtained from Hospital for Special Surgery (HSS) as part of the Accelerated Medicines Partnership. Cells were from male and female patients, ages 66–75. Cells were cultured in MEMα (Gibco) with 10% FBS and antibiotics. Experiments were performed on cells at passage 5–7.

Recombinant human or mouse IL-17A (PeproTech) was used at 100 ng/mL and human or mouse TNF (PeproTech) at 10 ng/mL. ST2 cells were treated with 500 nM NIK SMI1 (Sigma, SML3129) or 100-500 nM IKK inhibitor VII (Millipore, 401486) applied 18–24 hours prior to cytokine stimulation.

### Transwell chemotaxis assay.

WT or *Arid5a^–/–^* ST2 cells were pretreated with TNF (20 ng/mL) for 18 hours and then replated in α-MEM with 1% FBS. A standard transwell assay was performed with a 5 μm pore-sized transwell plate (Costar). PBMCs from C57BL/6 mice were cultured in the upper chamber in RPMI with 1% FBS for 5 hours. Cells in the bottom well were collected for flow cytometry. CountBright Plus Absolute Counting Beads (Invitrogen) were used to assess cell counts.

### qPCR.

RNA was isolated with RNeasy Mini Kits (Qiagen), and cDNA was synthesized by iScript cDNA Synthesis Kit (Bio-Rad). qPCR was performed with the SYBR Green FastMix ROX (Quanta Biosciences) on a CFX96 Real-Time PCR Detection System (Bio-Rad). Primers were from QuantiTect Primer Assays (Qiagen). Roadblock-qPCR was performed as described (24). Briefly, HK-2 cells were stimulated with 400 μM 4sU (Sigma). After 65**°**C denaturation, RNA was labeled by N-ethylmaleimide. cDNA was synthesized by SuperScript reverse transcriptase (Invitrogen) with oligo d(T) primers.

### RIP.

RIP was performed as described (68). HEK293T cells were cotransfected with human Arid5a-FLAG together with a Luc reporter fused to the murine *Ccl2*-3′ UTR. Extracts were isolated with lysis buffer (100 mM KCl, 5 mM MgCl_2_, 10 mM HEPES [pH 7.0], 0.5% NP-40, 1 mM dithiothreitol) with RNAse Out (100 U/mL, Invitrogen) and protease inhibitor cocktail (Sigma-Aldrich). Lysates were precleared with protein A agarose (Roche Applied Science) and subjected to RIP with anti-FLAG Abs (Sigma, F3165) or IgG isotype control (Cell Signaling, 5415), and *Luc* mRNA assessed by qPCR.

### Luciferase assays.

HK-2 or HEK293T cells were cotransfected with Arid5a-FLAG or EV control, the indicated *Luc* reporters fused to WT *Ccl2*-3′UTR, and a *Renilla* luciferase control reporter. After 24 hours, luciferase in cell lysates was assessed by GloMax Microplate Luminometer (Promega).

### Immunoblotting.

Western blotting was performed as described (12). Abs included IκBξ (Cell Signaling, 93726S), Arid5a (Invitrogen, P18112), C/EBPβ (Cell Signaling, 3082), C/EBPδ (Cell Signaling, 2318), β-actin (Abcam, ab49900), YY1 (Santa Cruz Biotechnology, sc1703), NF-κB1 p105/p50 (Cell Signaling, 13586S), NF-κB2 p100/p52 (Cell Signaling, 4882), TRAF2 (Santa Cruz Biotechnology, sc136999), IMP2 (Cell Signaling, 14672S), and MCPIP1 (R&D, MAB7875). Blots were visualized with a FluorChem E imager (ProteinSimple). Densitometry was performed by ImageJ.

### Immunofluorescence microscopy.

ST2 cells on glass slides were fixed in 4% paraformaldehyde and permeabilized in 0.1% Triton X-100. Cells were blocked in 5% goat serum with 0.3% Triton X-100 with 1% BSA and stained with DAPI, anti-RPL7A (15340-1-AP, Proteintech) or anti-Arid5a (P18112, Invitrogen) followed by fluorescence-conjugated secondary Abs. Slides were mounted using Antifade Mounting Medium (Vector Laboratories). Slides were visualized on a Nikon A1 confocal microscope, with image acquisition by NIS Viewer and quantification by NIS-Elements (Nikon).

### Flow cytometry.

Mouse synovia were digested with TM Research Grade Liberase (Sigma, 5401119001) in RPMI. Abs included CD45 (30-F11, Thermo Fisher), CD3 (BM10-37, BD Biosciences), CD4 (RM4-5, Thermo Fisher), CD11b (M1/70, BioLegend), CD11c (HL3, BD Biosciences), Ly6G (1A8, BD Biosciences), Ly6C (HK1.4, eBiosciences), F4/80 (BM8, eBioscience), RORγt (B2D, Invitrogen), and Tbet (4B10, Biolegend). Intracellular staining was performed with Cytofix/Cytoperm (BD) or FOXP3/ Staining Buffer Kit (Invitrogen). Dead cells were excluded using Ghost Dye (eBioscience). Data acquired with an LSR Fortessa and analyzed by FlowJo (TreeStar).

### Statistics.

Data were analyzed by GraphPad Prism. *P* < 0.05 was considered significant, determined by 2-tailed Student’s *t* test, 1-way ANOVA with appropriate Tukey’s, Dunnett’s, or Šídák’s multiple comparisons test, or 2-way ANOVA with Tukey’s multiple comparisons test. Each symbol represents an individual sample or mouse, as specified.

### Study approval.

Experiments complied with state and federal guidelines and protocols were approved by the University of Pittsburgh IACUC. The RA/OA FLS cells were provided as part of the Accelerated Medicines Partnership and the HSS FLARE team, approved by the HSS institutional IRB.

### Data availability.

The RA expression profiling array (17, 18) (accession no. GSE45291) was analyzed by GEO2R. DMARD-IR and TNF-IR groups were combined as the RA group (*n* = 20 for whole blood control, *n* = 493 for RA, *n* = 292 for SLE). T cell gene expression profiling (15, 16) (accession no. GSE118829) was analyzed by GEO2R (*n* = 63 not-treated, *n* = 63 for RA + MTX + IFX, *n* = 59 for RA + MTX, *n* = 66 for RA + TCZ, *n* = 11 for synovial fluid). Other data was from the Accelerating Medicines Partnership (AMP) RA Phase I project (20) (SDY998) and synovial Single-cell RNA-Seq (21) (SCP469, https://singlecell.broadinstitute.org). RELA, RELB, and NF-κB2 ChIP-Seq datasets from HEPG2 or GM12878 were from ENCODE and analyzed by IGV (version 2.17.4) for the human *ARID5A* proximal promoter (ENCSR164YJZ, ENCSR387QUV, ENCSR580HOI, ENCSR664POU).

## Author contributions

Conceptualization: SLG and YL. Methodology: PD, EL, and DPA. Investigation: YL, ID, SPV, DL, and AS. Resources: PD and AS. Writing: Original Draft: SLG and YL. Writing: Review and Editing: ID, SPV, EL, PD, and DPA. Visualization: YL. Supervision: SLG and PD. Funding Acquisition: SG.

## Funding support

This work is the result of NIH funding, in whole or in part, and is subject to the NIH Public Access Policy. Through acceptance of this federal funding, the NIH has been given a right to make the work publicly available in PubMed Central.

- Pittsburgh Foundation and NIH (AI162616, to SG). Pittsburgh Foundation and Boehringer-Ingelheim (to DA)
- Czech Science Foundation grant (24-12173S, to PD).

## Supplementary Material

Supplemental data

Unedited blot and gel images

Supporting data values

## Version 1 01/23/2026

Electronic publication
