# Supplementary material for: The RNA binding protein Arid5a is an activator of TNF signaling in rheumatoid arthritis
Source: JCI Insight. 2026 Jan 23;11(2):e196411. doi: 10.1172/jci.insight.196411 (PMC12892899; doi:10.1172/jci.insight.196411)
Supplement: Supplemental data [file jciinsight-11-196411-s097.pdf]

SUPPLEMENTARY MATERIALS

The RNA binding protein Arid5a is an activator of TNF signaling in rheumatoid arthritis

Yang Li, Ipsita Dey, Shachi P. Vyas, Alzbeta Synackova, Decheng Li, Erik Lubberts, Dana P. Ascherman, Peter Draber, Sarah L. Gaffen

Fig S1

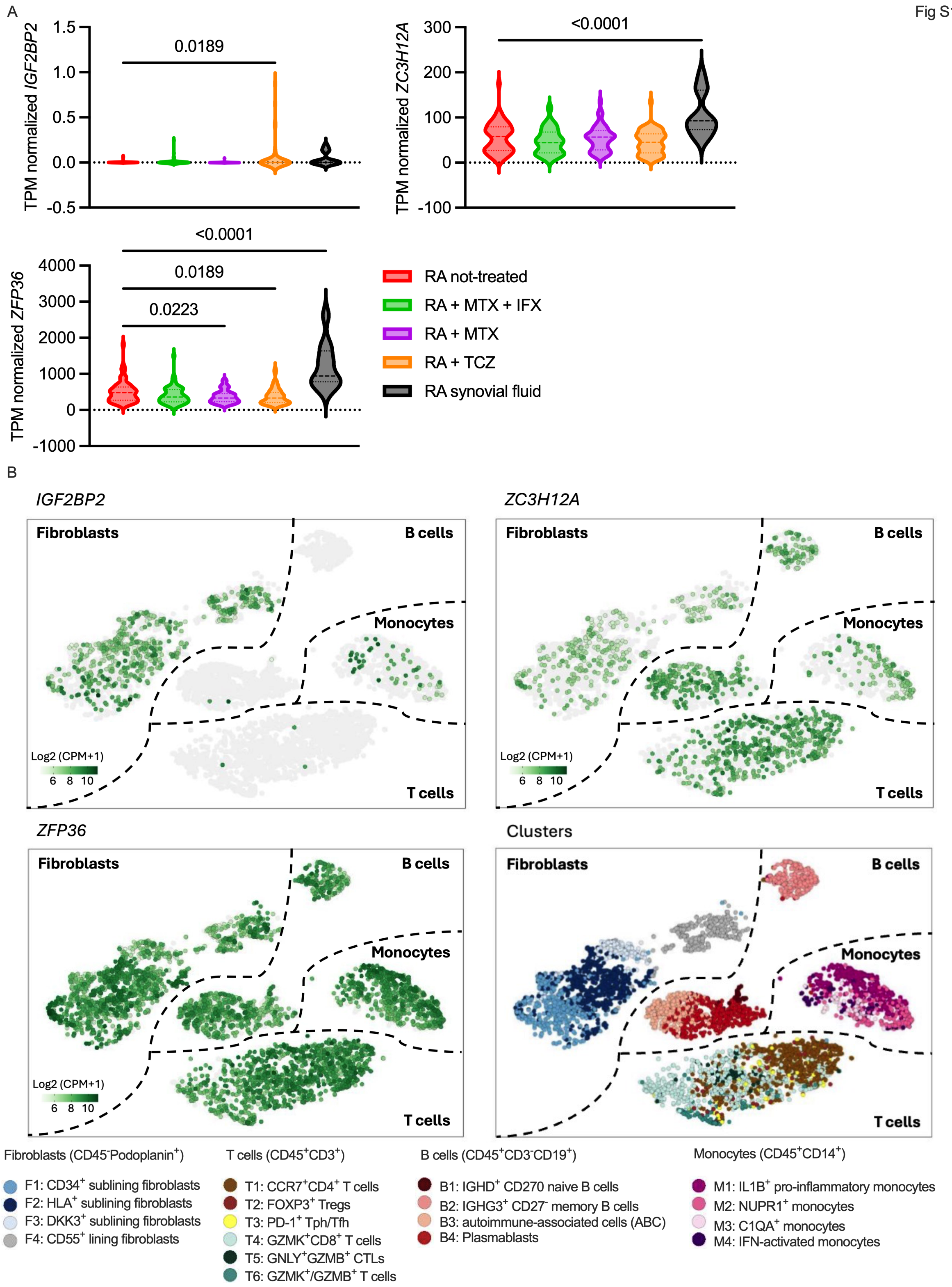

Fig S1 (associated with Fig. 1). RNA binding protein expression in human RA. A. RNA-Seq of data pooled from CD4$^+$ and CD8$^+$ groups treated with an anti-TNF biologic (infliximab, IFX),

methotrexate (MTX) or an anti-IL-6R (tocilizumab, TCZ). Levels in synovial fluid also shown (*15, 16*). Analyzed by one-way ANOVA with post-hoc Dunnett's test (n=63 for not-treated, n=63 for RA+IFX, n=59 for RA+MTX, n=66 for RA+TCZ, n=11 for synovial fluid), comparing each group to RA not-treated control. **B.** Single cell RNA-Seq data comparing osteoarthritis (OA) and RA (*20*)

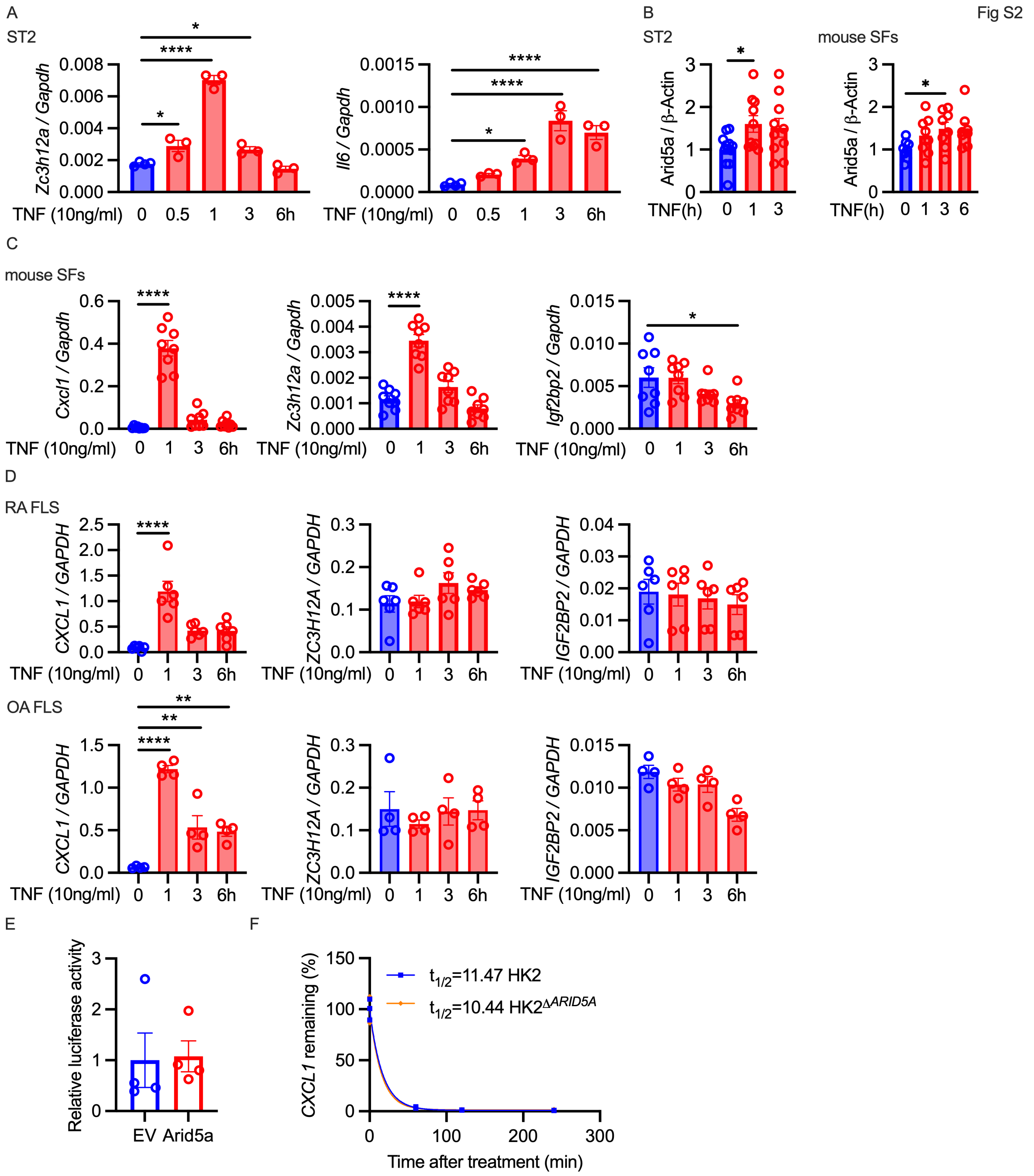

Fig S2 (associated with Fig. 2). RNA binding protein expression and function. A. ST2 cells were treated with TNF and expression of the indicated genes assessed by qPCR (n=3-4). Analyzed by one-way ANOVA with Dunnett's test for multiple comparisons, comparing each time point to the control (0 h). Data representative of 2 experiments. **B**. Densitometry for Fig 2b (n=10-12) and Fig 2d (n=8), analyzed by one-way ANOVA with Šídák's multiple comparisons test, comparing each time point to the control (0 h). Each symbol means one individual samples. **C**. Mouse primary

synovial fibroblasts were treated with TNF and mRNA expression assessed by qPCR (n=8). Analyzed by one-way ANOVA with Dunnett's test, comparing each time point to the 0 h control. Data pooled from 4 mice. **D**. RA and OA primary synovial fibroblasts were treated with TNF and mRNA assessed by qPCR (n=6 for RA, n=4 for OA). Analyzed by one-way ANOVA with Dunnett's test, comparing each time point to the 0 h control. Data pooled from 3 RA patients and 2 OA patients. **E**. HEK293T cells were transfected with Luc reporter and Arid5a or empty vector (EV). Analyzed by Student's t-test (n=4). Data representative of 2 experiments. **F**. Half-life of CXCL1 mRNA in HK2 or HK2$^{\Delta ARID5A}$ cells determined by 4sU labeling and decay assessment (n=3).

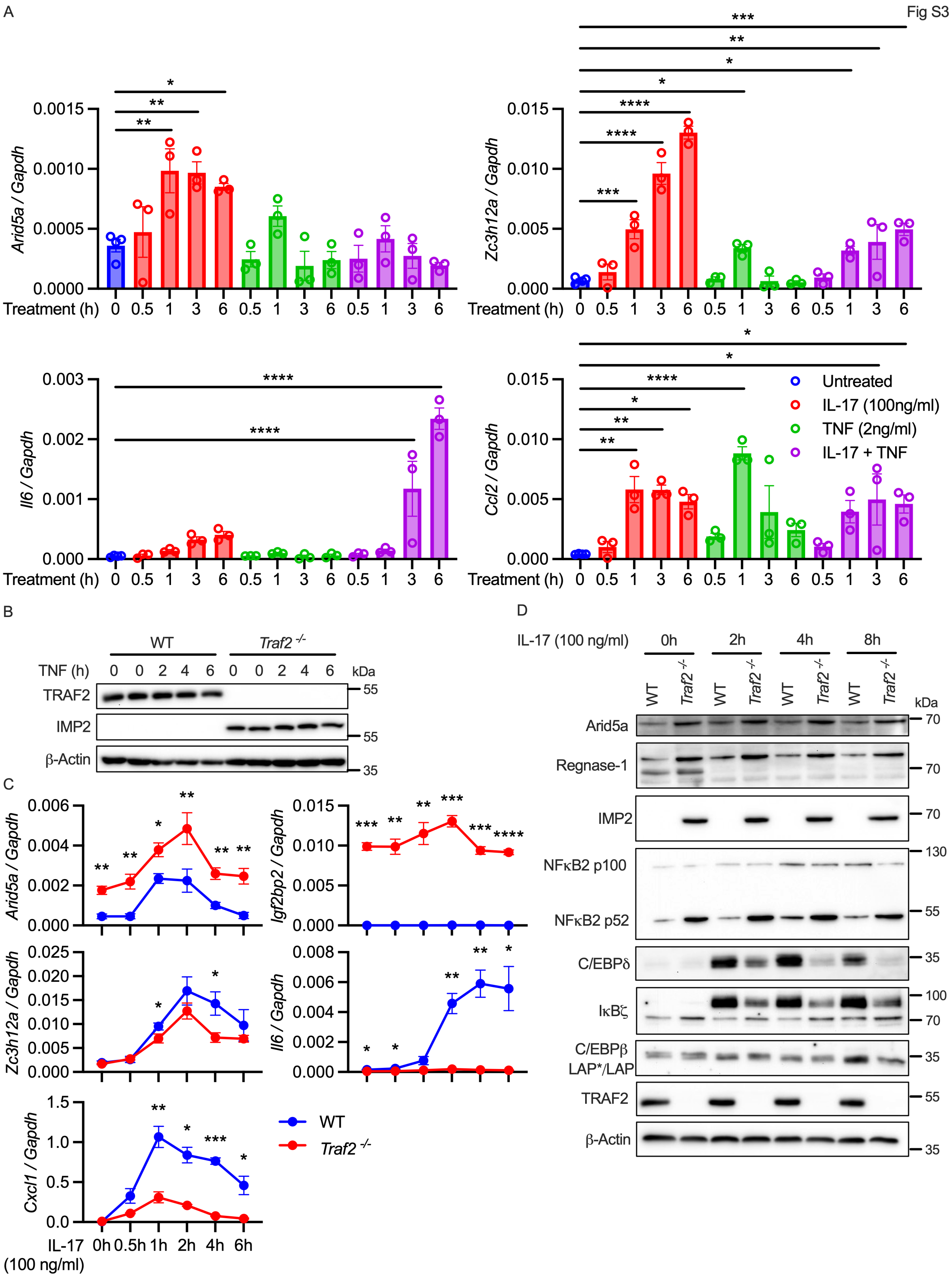

Fig S3 (associated with Fig. 3). TNF and IL-17 synergy. A. Indicated gene expression by qPCR

from ST2 cells treated with IL-17± TNF for the indicated times (n=3). Analyzed by one-way ANOVA with Dunnett's test, comparing each sample to 0 h control. Representative of 2 experiments. **B**. ST2 cell lysates were subjected to immunoblotting, representative of 2 experiments. **C**. WT or *Traf2*$^{-/-}$ ST2 cells were treated with IL-17 for the indicated times and mRNAs assessed by qPCR. Analyzed by Student's t-test for each time point (n=4), pooled from 2 independent experiments. **D**. WT or *Traf2*$^{-/-}$ ST2 cells were treated with IL-17 and lysates were immunoblotted for Arid5a, Regnase-1, IMP2, NFκB2, C/EBPδ, IκBζ, C/EBPβ, TRAF2 or β-Actin. Data representative of 2 experiments.

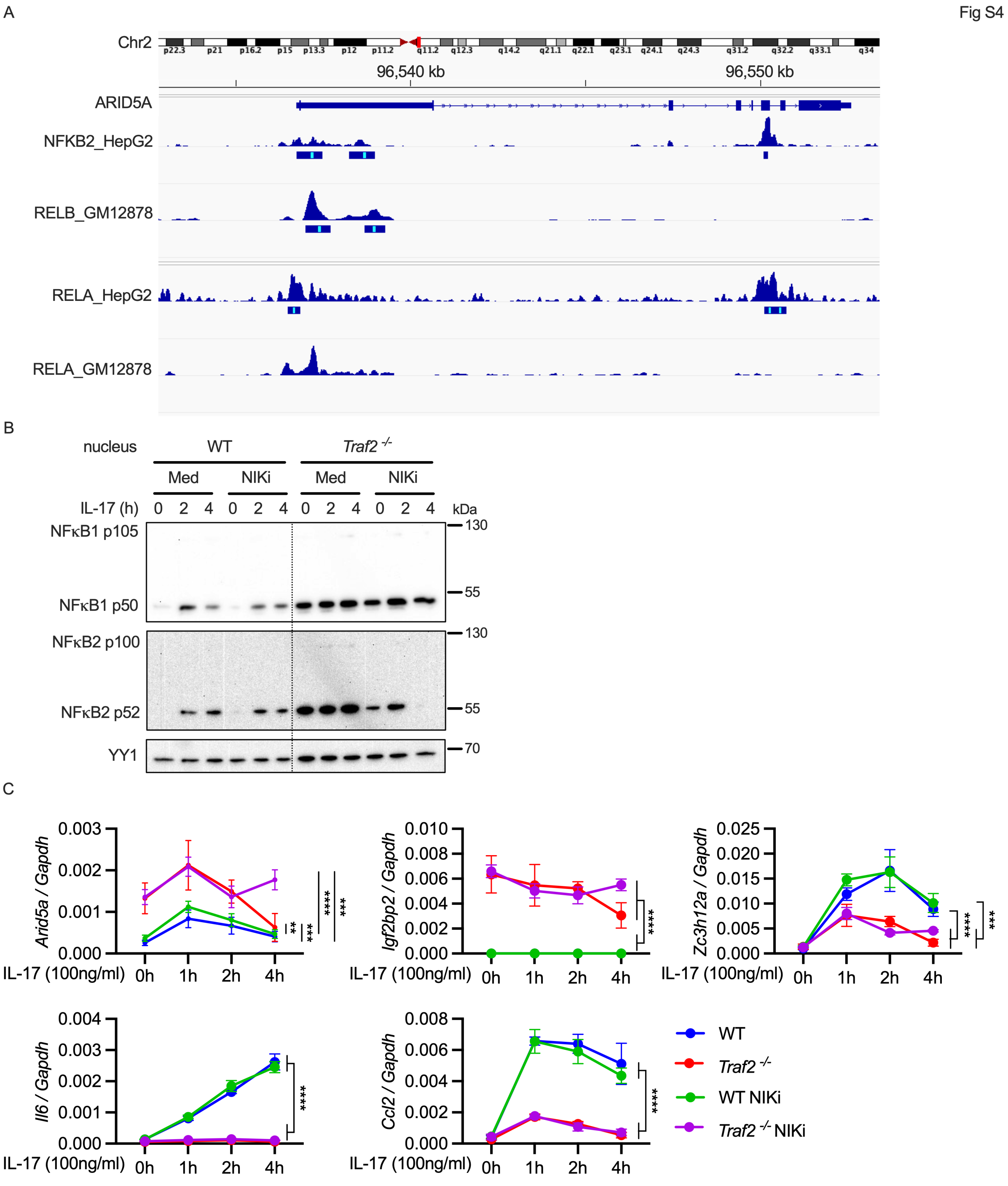

Fig S4 (associated with Fig. 4). NF-κB pathway regulation of Arid5a. A. NF-κB recognition sites in the ARID5A locus per ENCODE. WT or *Traf2*$^{-/-}$ ST2 cells were pretreated with NIK inhibitor (NIKi) for 16 h followed by IL-17 with NIKi for the indicated times. **B.** Lysates were immunoblotted for NFκB1, NFκB2 or YY1, representative of 2 experiments. **C.** Genes assessed by qPCR. Analyzed by two-way ANOVA with Tukey's test for multiple comparisons (n=3), representative of 3 experiments.

Fig S5

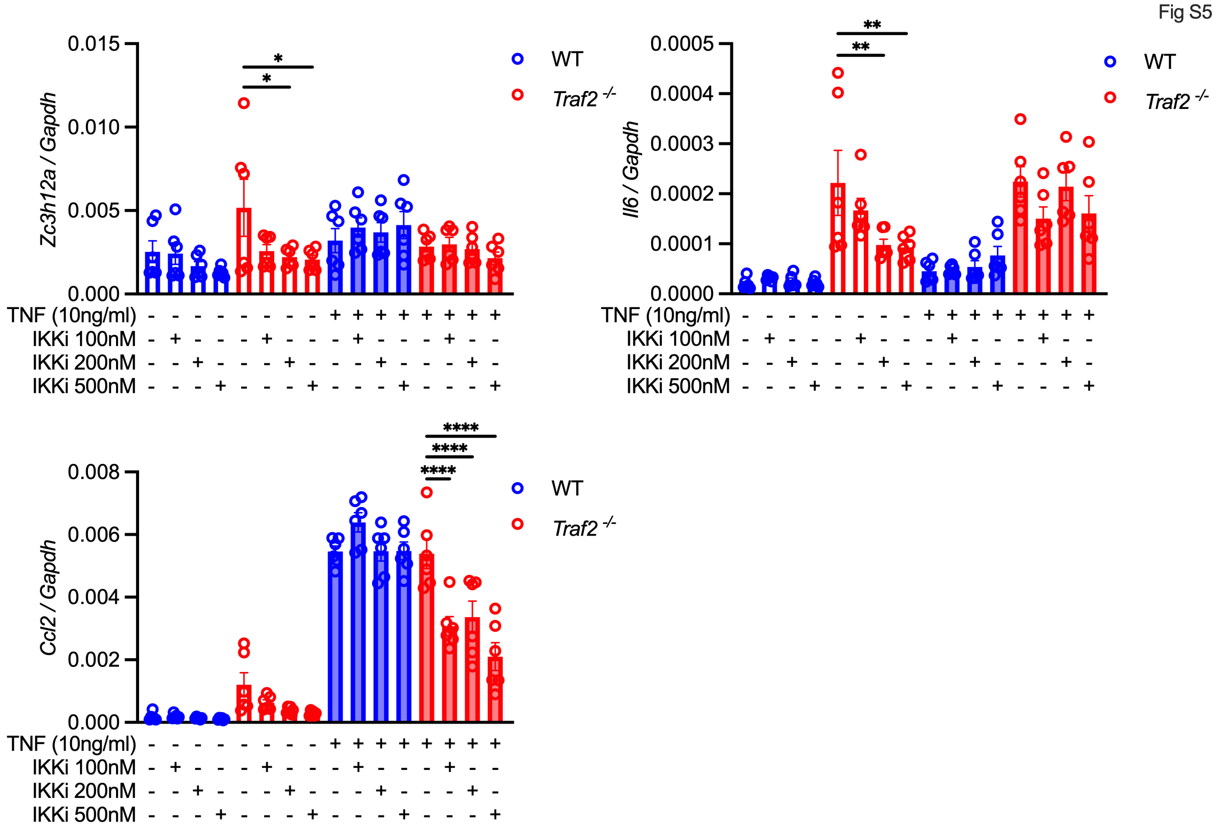

Fig S5 (associated with Fig. 4) IKK regulation of Traf2 signaling pathways. WT or *Traf2*$^{-/-}$ ST2 cells were pre-treated with IKK inhibitor (IKKi) for 20 h followed by TNF + IKKi for 1 h. Genes assessed by qPCR normalized to *Gapdh*. Analyzed by one-way ANOVA with Sidak's test for multiple comparisons between control and IKKi treated groups (n=6), representative of 3 experiments.

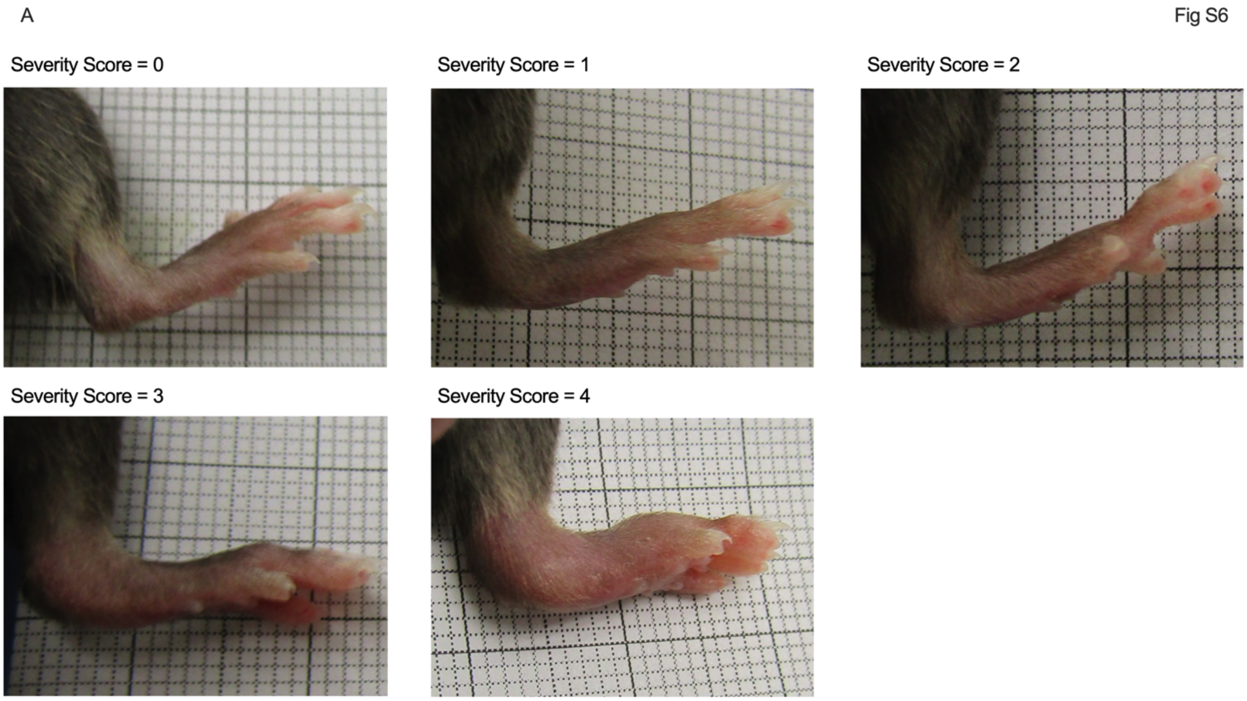

B

Severity score	Degree of inflammation
0	No evidence of erythema and swelling
1	Erythema and mild swelling confined to the tarsals or ankle joint
2	Erythema and mild swelling extending from the ankle to the tarsals
3	Erythema and moderate swelling extending from the ankle to metatarsal joints
4	Erythema and severe swelling encompass the ankle, foot and digits, or ankylosis of the limb

Fig S6 (associated with Fig 5). Severity scoring for CIA. A. Sample images (Day 48) of paws representing each severity score. **B.** Scale and criteria for scoring CIA. Adapted from Ref. (*43*).

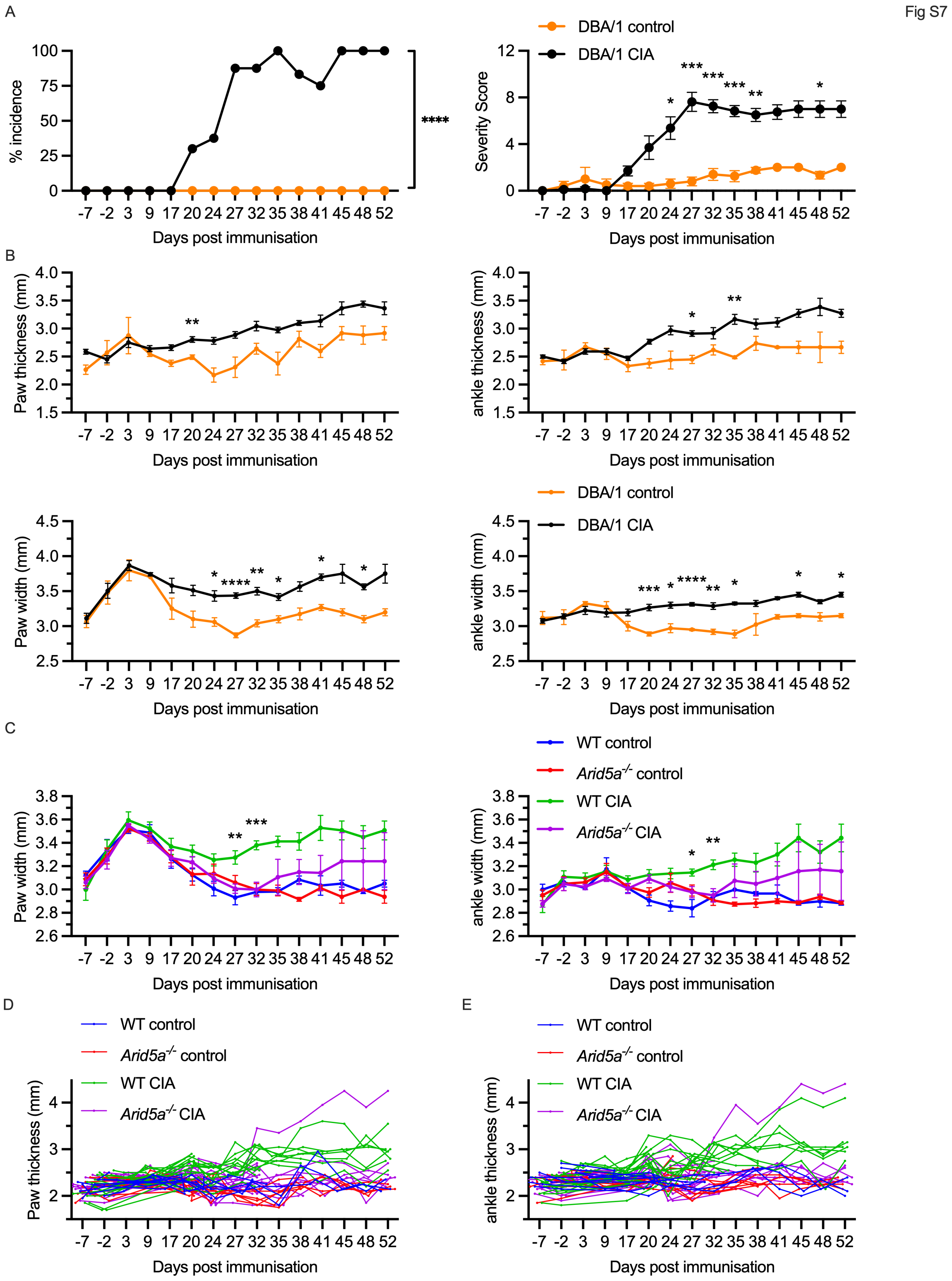

Fig S7. (associated with Fig 5). Arid5a drives CIA. A. Incidence and severity scores for DBA/1 mice. Incidence defined as total severity score of 6 or greater, analyzed by Student's t-Test.

Severity scores were analyzed by two-way ANOVA with Sidek's test for multiple comparisons per time point (DBA/1 control=5, DBA/1 CIA=10), pooled from 3 independent experiments. **B.** paw and ankle thickness or width of DBA/1 mice. **C.** paw and ankle width of C57BL/6 mice. Data analyzed as the described in Fig 5. Data pooled from 3 independent experiments (WT control=8, WT CIA=15, *Arid5a*$^{-/-}$ control=8, *Arid5a*$^{-/-}$ CIA=18). **D-E.** Paw and ankle thickness in WT (C57BL/6) and *Arid5a*$^{-/-}$ mice (same data as Fig 5d-e). Each line indicates one individual mice (WT control=8, WT CIA=15, *Arid5a*$^{-/-}$ control=8, *Arid5a*$^{-/-}$ CIA=18), pooled from 3 independent experiments.

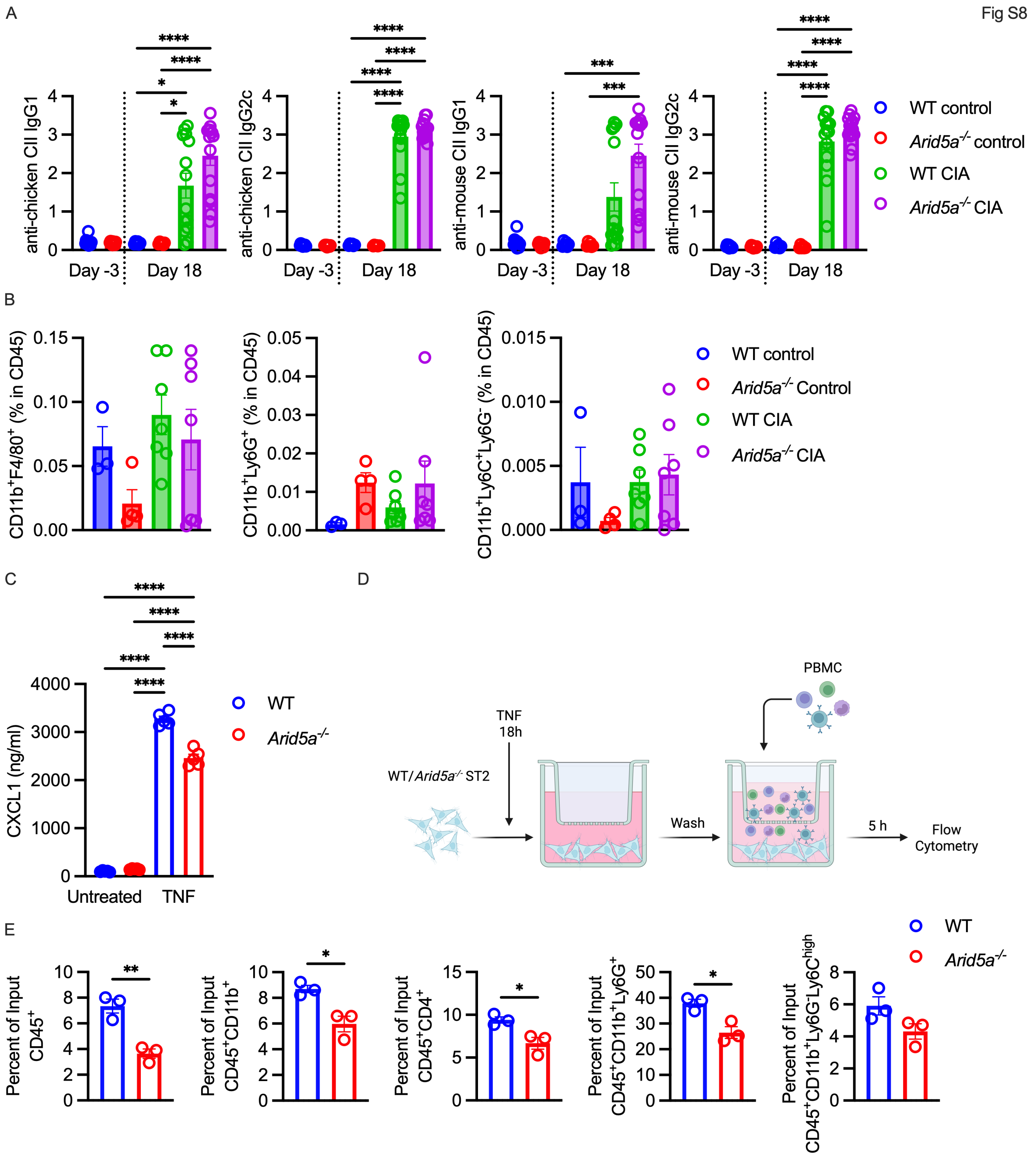

Fig S8. (associated with Fig 6). Arid5a enhances the chemotaxis of inflammatory cells. **A.** Serum levels of anti-chicken and anti-mouse collagen-II. Data pooled from 3 independent experiments (n=6-15). Analyzed by one-way ANOVA with Tukey's test. **B.** Percentages of $CD11b^+F4/80^+$, $CD11b^+Ly6G^+$ and $CD11b^+Ly6C^+Ly6G^-$ in $CD45^+$ cells in synovial samples. Analyzed by one-way ANOVA with Tukey's test (n=3-7). **C.** WT or *Traf2*$^{-/-}$ ST2 cells were treated with TNF for 18 h and CXCL1 in supernatants determined by ELISA. Analyzed by one-way ANOVA with Tukey's test (n=5). **D-E.** WT or *Arid5a*$^{-/-}$ ST2 cells were pretreated with TNF (20 ng/ml) for 18 h and PBMC migration determined by transwell assay. PBMCs isolated from C57BL/6 WT mice. Analyzed by Student's t-test (n=3).
