# Supplementary material for: The RNA binding protein Arid5a is an activator of TNF signaling in rheumatoid arthritis
Source: JCI Insight. 2026 Jan 23;11(2):e196411. doi: 10.1172/jci.insight.196411 (PMC12892899; doi:10.1172/jci.insight.196411)

Full unedited gel for Fig 2b

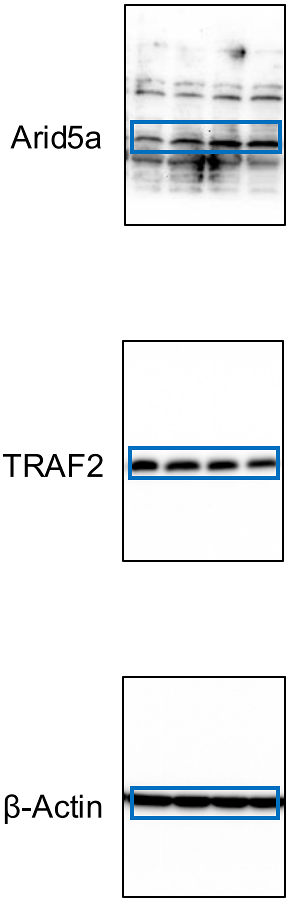

Full unedited gel for Fig 2d

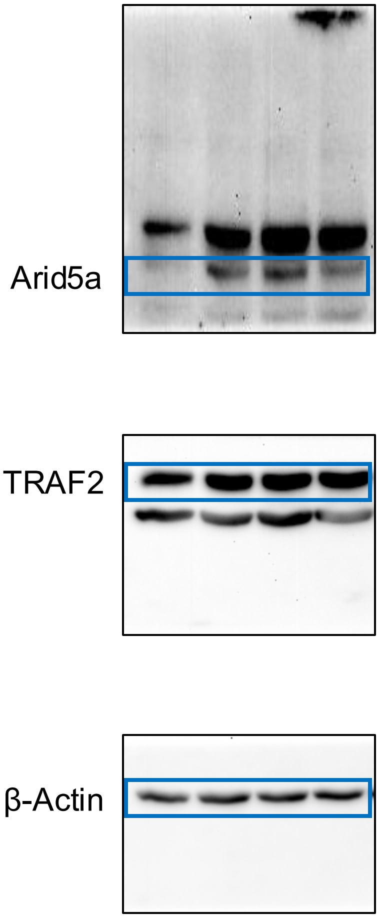

Full unedited gel for Fig 2f

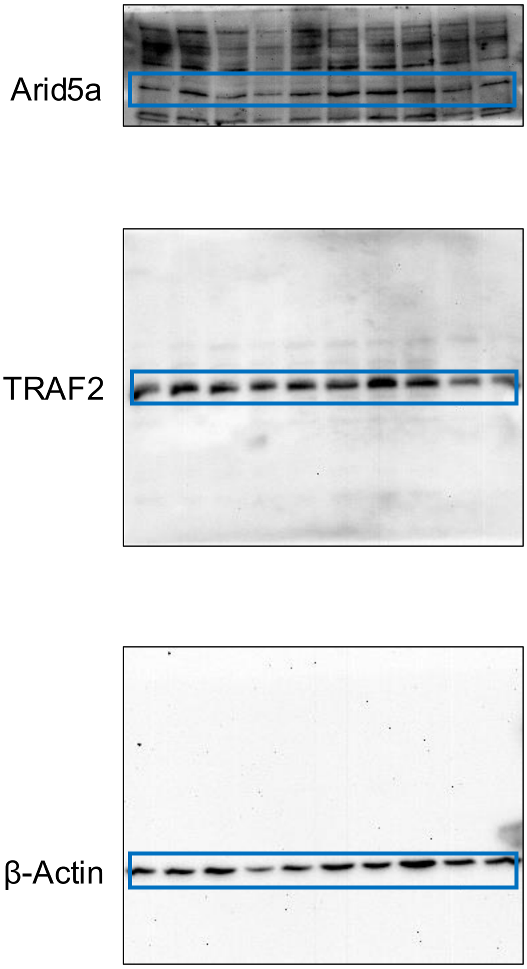

Full unedited gel for Fig 3b

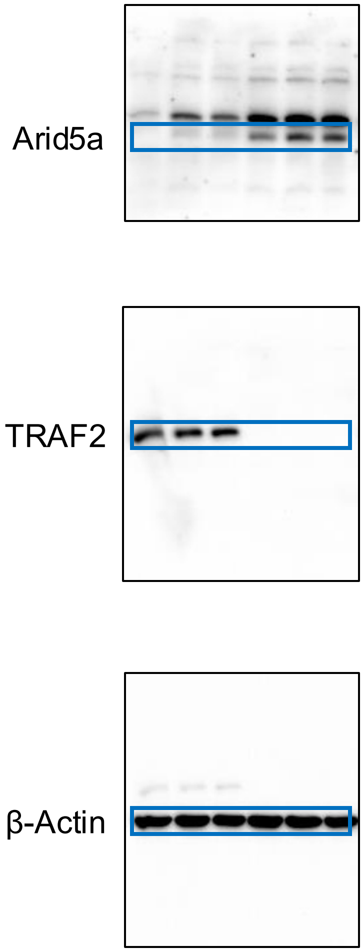

Full unedited gel for Fig 4a

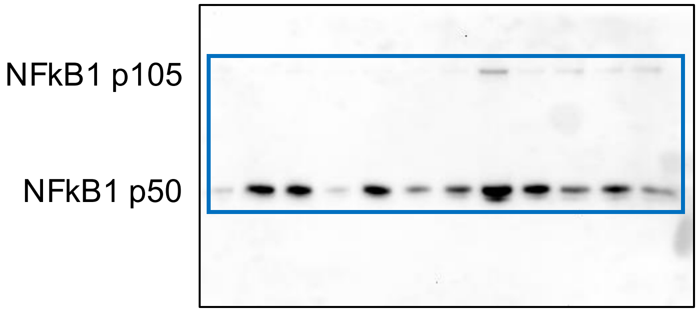

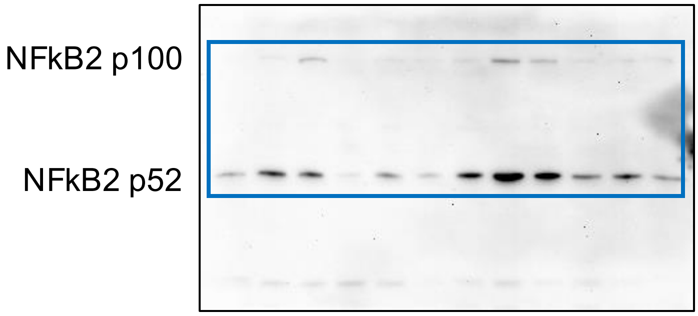

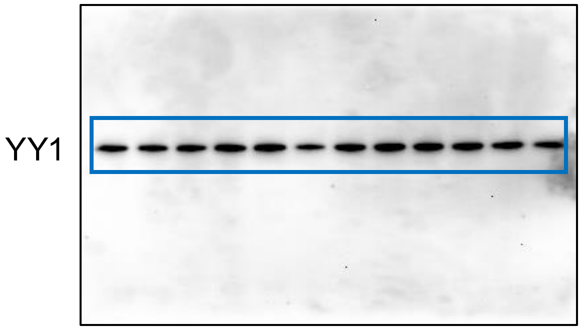

Full unedited gel for Fig S3b

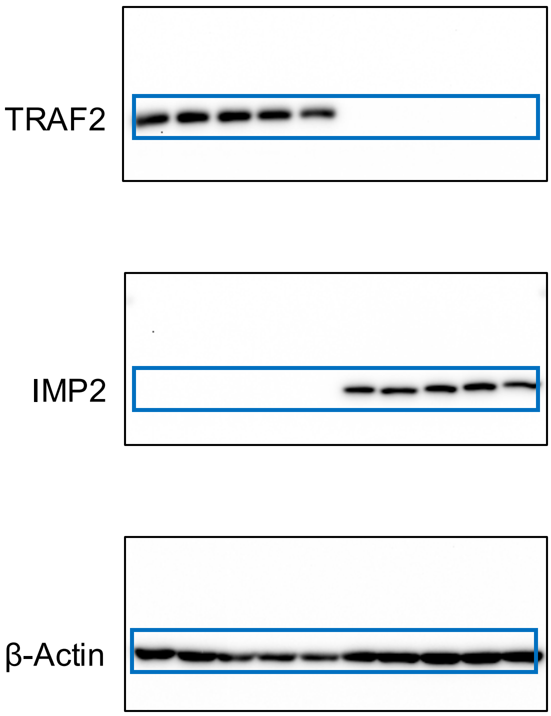

Full unedited gel for Fig S3d

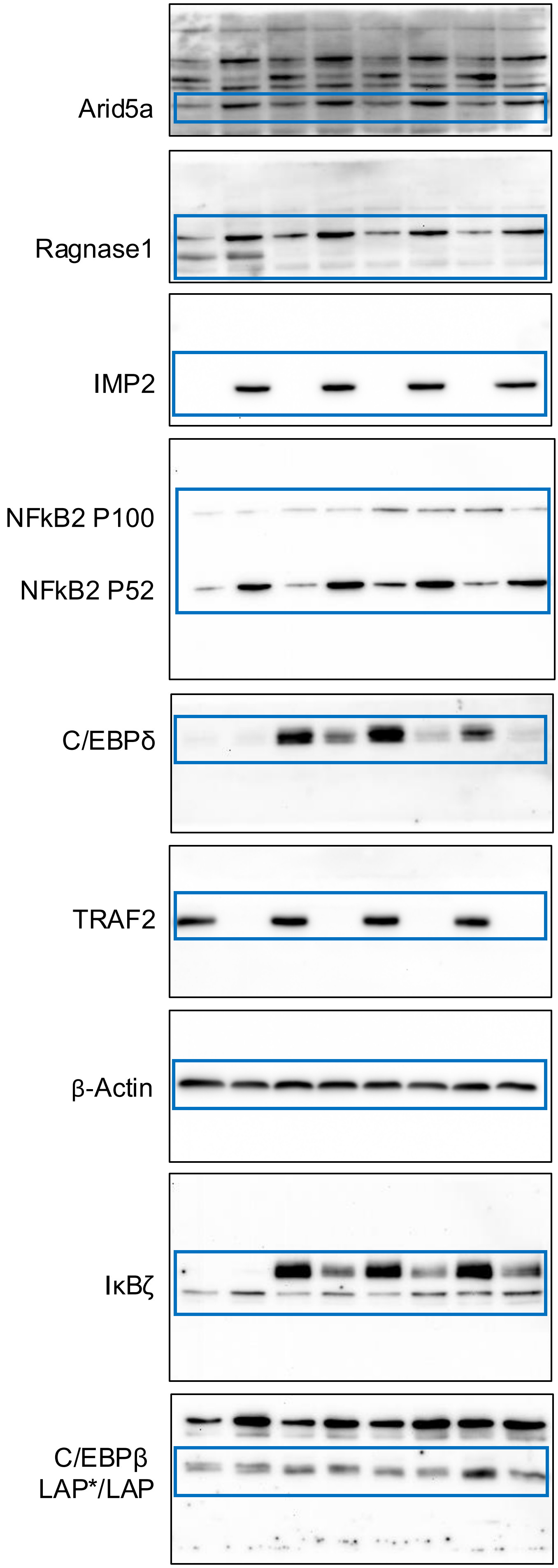

Full unedited gel for Fig S4b

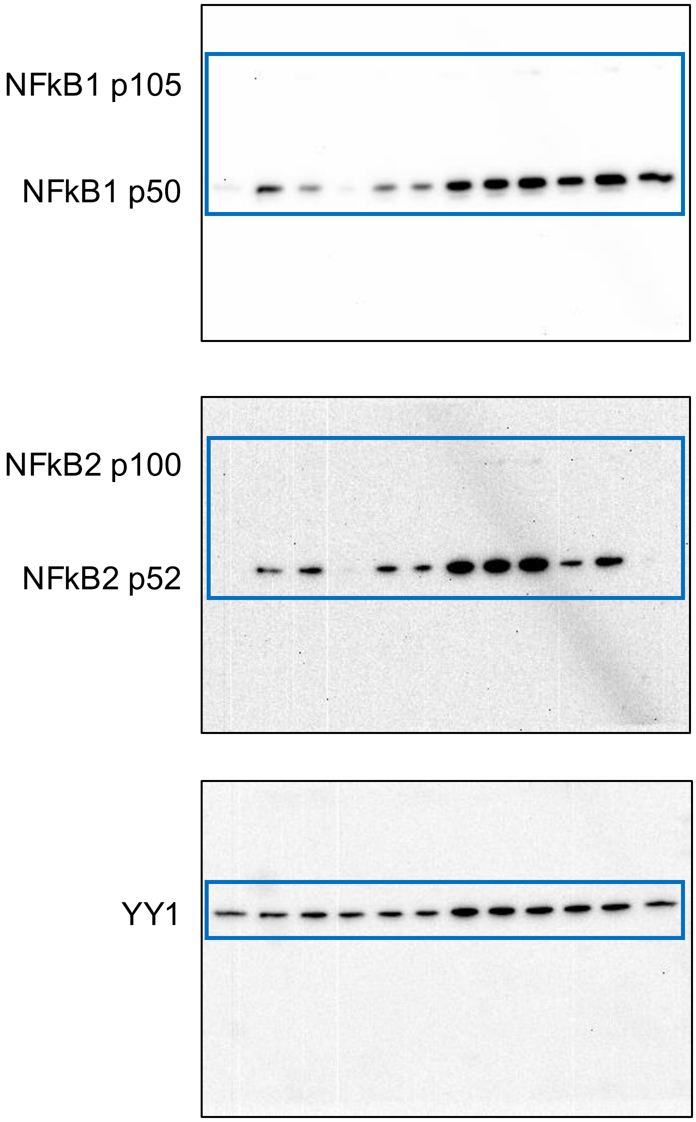

Supplement: Unedited blot and gel images [file jciinsight-11-196411-s098.pdf]
